# Diphenyl Ethers from a Marine-Derived *Aspergillus sydowii*

**DOI:** 10.3390/md16110451

**Published:** 2018-11-16

**Authors:** Ya-Nan Wang, Yan-Hua Mou, Yu Dong, Yan Wu, Bing-Yu Liu, Jian Bai, Dao-Jiang Yan, Le Zhang, Dan-Qing Feng, Yue-Hu Pei, You-Cai Hu

**Affiliations:** 1 School of Life Science and Biopharmaceutics, Shenyang Pharmaceutical University, Shenyang 110016, China; mu_hua_jj@sina.com (Y.-H.M.); allenn7@foxmail.com (Y.D.); 2 State Key Laboratory of Bioactive Substance and Function of Natural Medicines, Institute of Materia Medica, Chinese Academy of Medical Sciences and Peking Union Medical College, Beijing 100050, China; wangyanan@imm.ac.cn (Y.-N.W.); wuyan@imm.ac.cn (Y.W.); liubingyu@imm.ac.cn (B.-Y.L.); baijian@imm.ac.cn (J.B.); yandj@imm.ac.cn (D.-J.Y.); zhangle@imm.ac.cn (L.Z.); 3 State-Province Joint Engineering Laboratory of Marine Bioproducts and Technology, College of Ocean & Earth Sciences, Xiamen University, Xiamen 361102, China; dqfeng@xmu.edu.cn

**Keywords:** *Aspergillus sydowii*, fungal natural product, diphenyl ethers, structure elucidation, cytotoxicity

## Abstract

Six new diphenyl ethers (**1**–**6**) along with eleven known analogs were isolated from the ethyl acetate extract of a marine-derived *Aspergillus sydowii* guided by LC-UV-MS. Their structures were unambiguously characterized by HRESIMS, NMR, as well as chemical derivatization. Compounds **1** and **2** are rare diphenyl ether glycosides containing d-ribose. The absolute configuration of the sugar moieties in compounds **1**–**3** was determined by a LC-MS method. All the compounds were evaluated for their cytotoxicities against eight cancer cell lines, including 4T1, U937, PC3, HL-60, HT-29, A549, NCI-H460, and K562, and compounds **1**, **5**, **6**, and **8**–**11** were found to exhibit selective cytotoxicity against different cancer cell lines.

## 1. Introduction

**Figure 1:**
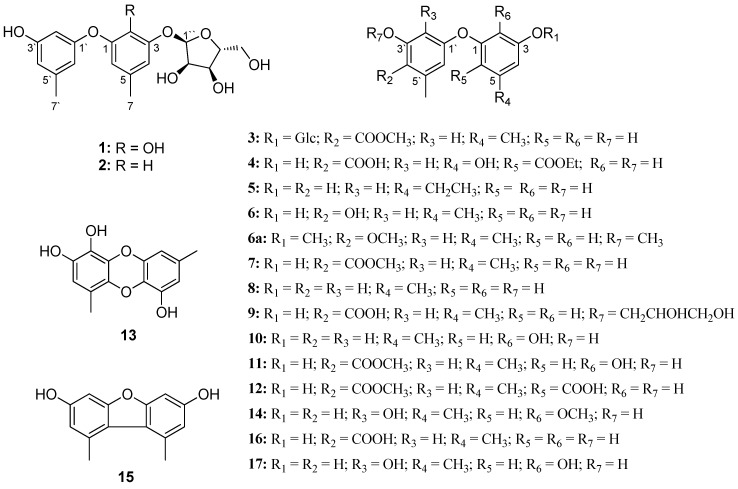
Structures of compounds **1**–**17** and **6a**.

Marine microorganisms have become an important source of pharmacologically active metabolites [1,2,3,4,5]. In particular, marine-derived fungi have been identified as promising producers of chemically and biologically diverse natural products [6]. The genus *Aspergillus*, with over 200 species, has attracted considerable attention as a rich source of bioactive compounds including polyketides, peptides, terpenoids [7,8,9] and others. Diphenyl ethers are a group of polyketides with simple structures that are widely distributed in various species of *Aspergillus* [10,11,12,13] and have been reported to show significant diverse bioactivities, such as antiviral [14], antimicrobial [14,15], *β*-glucuronidase enzyme inhibitory [16], radical-scavenging [17], cytotoxicity [10,14,18,19,20,21,22,23,24], anti-Aβ 42 aggregation [24], regulating actin function [25] and phytocidal activities [26]. The cytotoxicity of diphenyl ethers against a variety of cancer cell lines has been widely reported. For example, diorcinol, cordyol C, and 3,7-dihydroxy-1,9-dimethyldibenzofuran showed cytotoxicity against HeLa and DU145 cell lines with IC_50_ values of 1.20–7.12 μM [22]. Sinopestalotiollide D showed cytotoxicity against HeLa, HCT 116 and A549 cell lines with IC_50_ values of 1.19, 2.66 and 2.14 μM, respectively [19]. In order to further discover novel cytotoxic diphenyl ethers from natural sources, a marine-derived fungus *Aspergillus sydowii* strain FNA026 was investigated. As a result, seventeen diphenyl ethers **1**–**17** (Figure 1), including six new ones (**1**–**6**) were obtained from the ethyl acetate extract of *A. sydowii* guided by LC-UV-MS. Among them, compounds **1**–**3** were identified as diphenyl ether glycosides which are rare in marine secondary metabolites. Herein, we report the isolation, structure elucidation and cytotoxicities of the isolated compounds**.**

## 2. Results and Discussion

**Figure 2:**
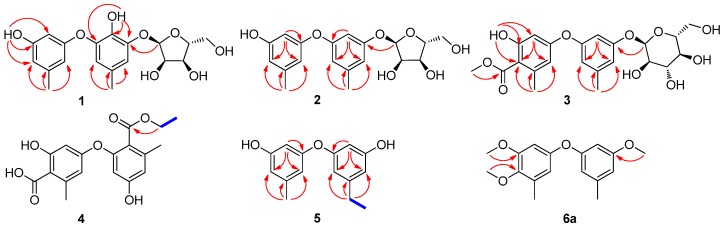
Key ^1^H-^1^H COSY (blue lines) and key HMBC (arrows) correlations of **1**–**5** and **6a**.

Compound **1** was obtained as colorless oil. Its IR spectrum suggested the presence of hydroxy (3320 cm^−1^) and aromatic (1596, 1512, and 1464 cm^−1^) groups. The molecular formula of **1** was established as C_19_H_22_O_8_ on the basis of HRESIMS, which gave a sodium adduct ion at *m*/*z* 401.1206 [M + Na]^+^. The ^1^H NMR spectrum (in DMSO-*d*_6_) of **1** exhibited signals for two methyl groups at *δ*_H_ 2.15 (3H, s), 2.18 (3H, s), five aromatic protons at *δ*_H_ 6.02 (1H, brs), 6.12 (1H, brs), 6.21 (1H, brs), 6.46 (1H, brs) and 6.81 (1H, brs), as well as two phenolic hydroxy groups at *δ*_H_ 8.41 (1H, s) and 9.26 (1H, s). Analysis of its ^13^C NMR and HSQC spectra (see Supplementary Information Figures S4 and S6) indicated the presence of five sp^2^ methines at *δ*_C_ 100.3, 107.5, 109.6, 112.9, and 115.9, seven sp^2^ quaternary carbons at *δ*_C_ 127.3, 137.3, 139.5, 142.0, 145.7, 158.2, and 159.1 and two methyl carbons at *δ*_C_ 20.6 and 21.2. Careful analysis of the ^1^H NMR and ^13^C NMR data (Table 1 and Table 2) of **1** indicated that it was a diphenyl ether derivative with one tetra-substituted phenyl and one trisubstituted phenyl groups, as well as an additional pentose residue. The aglycone of **1** was identified as cordyol C [14] by comparison of the 1D NMR data of **1** with those of cordyol C and was supported by the key HMBC correlations from H_3_-7 (*δ*_H_ 2.18) to C-4 (*δ*_C_ 112.9), C-5 (*δ*_C_ 127.3), and C-6 (*δ*_C_ 115.9), and from 2-OH (*δ*_H_ 8.41) to C-1 (*δ*_C_ 142.0), C-2 (*δ*_C_ 137.3) and C-3 (*δ*_C_ 145.7), from 3′-OH (*δ*_H_ 9.26) to C-2′ (*δ*_C_ 100.3), C-3′ (*δ*_C_ 158.2), and C-4′ (*δ*_C_ 109.6), as well as from H_3_-7′ (*δ*_H_ 2.15) to C-4′ (*δ*_C_ 109.6), C-5′ (*δ*_C_ 139.5), and C-6′ (*δ*_C_ 107.5). The pentose residue in **1** showed signals at *δ*_C_ 101.3, 72.3, 69.4, 86.7 and 61.6 in its ^13^C NMR spectrum and an anomeric proton *δ*_H_ 5.50 (1H, d, *J* = 4.6 Hz) in its ^1^H NMR spectrum, which suggested the presence of an *α*-ribofuranosyl moiety [27]. The key HMBC correlation from H-1″ (*δ*_H_ 5.50) to C-3 (*δ*_C_ 145.7) (Figure 2) established the connection between the ribose and the diphenyl ether moiety. The *α*-ribose was determined to have a d-configuration by comparison in LC-MS of the retention time of the thiocarbamoyl-thiazolidine derivative prepared after hydrolisis of **1** with those obtained from d-ribose and l-ribose standards (see Supplementary Information Figure S54). Thus, compound **1** was characterized as cordyol C-3-*O*-*α*-d-ribofuranoside.

Compound **2** was isolated as colorless oil. Its molecular formula was determined to be C_19_H_22_O_7_, having one oxygen less than that of **1**, on the basis of HRESIMS data. The detailed analysis of the ^1^H NMR and ^13^C NMR data (Table 1 and Table 2) of **2** indicated that it had a diphenyl ether moiety with two trisubstituted benzene rings, and an additional pentose residue. The two trisubstituted rings were assigned by the HMBC correlations (Figure 2) from H_3_-7 (*δ*_H_ 2.27) to C-4 (*δ*_C_ 111.8), C-5 (*δ*_C_ 141.6), C-6 (*δ*_C_ 112.1) and from H-2 (*δ*_H_ 6.20) to C-1 (*δ*_C_ 159.6), C-4 (*δ*_C_ 111.8), C-6 (*δ*_C_ 112.1); from H_3_-7′ (*δ*_H_ 2.22) to C-4′ (*δ*_C_ 114.2), C-5′ (*δ*_C_ 141.7), C-6′ (*δ*_C_ 113.5) and H-2′ (*δ*_H_ 6.57) to C-1’ (*δ*_C_ 159.7), C-4′ (*δ*_C_ 114.2), C-6′ (*δ*_C_ 113.5). The anomeric proton signal at *δ*_H_ 5.57 (1H, d, *J* = 4.5 Hz) in its ^1^H NMR spectrum, and carbon signals at *δ*_C_ 102.3, 73.4, 71.2, 87.5 and 63.2 in its ^13^C NMR spectrum suggested the pentose residue in **2** was an *α*-ribofuranosyl. The sugar moiety in **2** was identified as d-ribose by using the same method as described for **1** (see Supplementary Information Figure S55). The key HMBC correlation from H-1″ (*δ*_H_ 5.57) to C-3 (*δ*_C_ 159.5) (Figure 2) established the connection between the ribose and the diphenyl ether moiety. As a result, compound **2** was determined as diorcinol-3-*O*-*α*-d-ribofuranoside.

The molecular formula C_22_H_26_O_10_ of compound **3** was deduced from positive HRESIMS which gave a sodium adduct ion at *m*/*z* 473.1403 [M + Na]^+^. Its ^13^C NMR spectrum (Table 2) displayed 22 carbon resonances, including 6 signals for a hexose residue at *δ*_C_ 97.9, 71.5, 73.0, 69.8, 73.8 and 60.6, suggesting **3** to be a glucoside [28]. The ^1^H and ^13^C NMR spectra for **3** (Table 1 and Table 2) indicated the aglycone in **3** as 4-methoxycarbonyl diorcinol [8], which was supported by key HMBC correlations from H_3_-7 (*δ*_H_ 2.26) to C-4 (*δ*_C_ 113.5), C-5 (*δ*_C_ 140.5), C-6 (*δ*_C_ 113.6) and from H-2 (*δ*_H_ 6.57) to C-4 (*δ*_C_ 113.5), C-6 (*δ*_C_ 113.6), from H_3_-7′ (*δ*_H_ 2.21) to C-4′ (*δ*_C_ 114.9), C-5′ (*δ*_C_ 139.1), C-6′ (*δ*_C_ 110.7), from H-2′ (*δ*_H_ 6.28) to C-4′ (*δ*_C_ 114.9), C-6′ (*δ*_C_ 110.7), and from 3′-O*H* (*δ*_H_ 10.26) to C-2′ (*δ*_C_ 102.6), C-3′ (*δ*_C_ 157.6), C-4′ (*δ*_C_ 114.9), as well as from H_3_-9′ (*δ*_H_ 3.78) to C-8′ (*δ*_C_ 168.5). The anomeric signal at *δ*_H_ 5.35 (1H, d, *J* = 3.6 Hz)/*δ*_C_ 97.9 (C-1″) and the one-bond coupling constant of 172.9 Hz between C-1″ and H-1″ (see Supplementary Information Figure S23) supported an *α*-configuration of the *O*-glucoside [29]. The absolute configuration of glucose moiety in **3** was determined as d-glucose based on LC-ESI-MS analysis (see Supplementary Information Figure S56). The key HMBC correlation from H-1″ (*δ*_H_ 5.35) to C-3 (*δ*_C_ 158.3) (Figure 2) established the connection between the glucose and diphenyl ether moiety. Thus, the structure of compound **3** was determined to be 4-methoxycarbonyl diorcinol-3-*O*-*α*-d-glucoside.

Compound **4** was obtained as colorless oil. Its molecular formula was determined to be C_18_H_18_O_7_ by HRESIMS, having a fragment of C_3_H_4_O_2_ more than that of 4-carboxydiorcinal (**16**). The ^1^H and ^13^C NMR spectra for **4** (Table 1 and Table 2) and **16** showed very similar signals, with the exception that H-6 at *δ*_H_ 6.17 for **16** was missing for **4**, which indicated that the CH at C-6 in **16** was replaced by another substitution in **4**. Analysis of the ^1^H NMR and ^13^C NMR (Table 1 and Table 2) and ^1^H-^1^H COSY correlation data (Figure 2) of **4** indicated the presence of an ester carbonyl (C-8) at *δ*_C_ 172.4 and an oxygenated ethyl group (C-9/C-10) at *δ*_C_ 62.5 and *δ*_C_ 14.5. The presence of an ethyl ester unit was confirmed by the HMBC correlation from H_2_-9 (*δ*_H_ 4.40) to C-8 (*δ*_C_ 172.4) (Figure 2). Thus, the structure of **4** was elucidated as 2-(ethoxycarbonyl)-4′-carboxydiorcinal.

The HRESIMS of **5** displayed a protonated ion [M + H]^+^ at *m*/*z* 245.1183, corresponding to the molecular formula of C_15_H_16_O_3_, one more carbon and two more hydrogens than that of diorcinol (**8**). The ^1^H and ^13^C NMR spectra for **5** (Table 1 and Table 2) and **8** showed very similar signals, with the exception that a methyl at C-7 (*δ*_C_ 21.5) in **8** was substituted by an ethyl group in **5**, which was confirmed by HMBC correlations from the ethyl protons at H_2_-7 (*δ*_H_ 2.53) to C-4 (*δ*_C_ 111.7), C-5 (*δ*_C_ 148.2), and C-6 (*δ*_C_ 110.8). The full structure of **5** was further confirmed by COSY and HMBC experiments (Figure 2). As a result, **5** was elucidated as 7-ethyldiorcinol.

The molecular formula C_14_H_14_O_4_ of **6** was determined by negative HRESIMS at *m*/*z* 245.0808 [M − H]^−^, one more oxygen than those of **8**. The ^1^H and ^13^C NMR spectra for **6** (Table 1 and Table 2) showed very similar signals to those of **8**, with the exception that the tertiary carbon signal at *δ*_C_ 111.8 for **8** was replaced by a quaternary carbon signal at *δ*_C_ 140.8 for **6**. These data indicated that the hydrogen at C-6 (*δ*_C_ 111.8) in **8** was substituted by a hydroxy group in **6**. The two hydroxy groups in the B ring did not display any correlation in the HMBC spectrum, and therefore the position of the ether linkage between the two benzene rings could not be determined by 2D NMR data at this stage. Therefore, full methylation of **6** was completed with CH_3_I/K_2_CO_3_, which afforded its derivative **6a**. HMBC correlations of **6a** from H_3_-8 (*δ*_H_ 3.73) to C-3 (*δ*_C_ 162.3), from H_3_-8′ (*δ*_H_ 3.78) to C-3′ (*δ*_C_ 154.8) and from H_3_-9′ (*δ*_H_ 3.75) to C-4′ (*δ*_C_ 144.5) (Figure 2) demonstrated that the three free hydroxy groups of **6** were attached to C-3, C-3′ and C-4′, respectively. Therefore, **6** was unambiguously determined as 3-hydroxydiorcinol.

The eleven known compounds (**7**–**17**) were identified as 4-methoxycarbonyl diorcinol (**7**) [10], diorcinol (**8**) [11], glyceryl diorcinolic acid (**9**) [12], cordyol C (**10**) [14], aspergilol E (**11**) [13], 4-hydroxy-2-(3′-hydroxy-4-methoxycarbonyl-5′-methylphenoxy)-6-methylbenzoic acid (**12**) [16], gibellulin B (**13**) [11], diorcinols F (**14**) [30], 3,7-dihydroxy-1,9-dimethyldibenzofuran (**15**) [31], 4-carboxydiorcinal (**16**) [32] and aspermutarubrol (**17**) [33] by comparison of their spectroscopic data to those reported in the literature.

The cytotoxicity of all the isolated compounds was tested against a series of cancer cell lines, including 4T1 (Mouse Breast Cancer cell line), U937 (Human Histiocytic Lymphoma cell line), PC3 (Human Prostate Cancer cell line), HL-60 (Human Leukemia cell line), HT-29 (Human Colorectal Adenocarcinoma cell line), A549 (Human Lung Adenocarcinomic cell line), NCI-H460 (Human Large Cell Lung Cancer cell line) and K562 (Human Myelogenous Leukemia cell line), with doxorubicin (DOX) as positive control (Table 3). None of the compounds showed cytotoxicity against the five solid cancer cell lines (4T1, PC3, HT-29 and NCI-H460). Compounds **1**, **5**, **8** and **9** showed moderate cytotoxicity against A549. These results suggested that glycosylation of the 3-hydroxy group seems to negatively contribute to its cytotoxicity against A549 cell line (**2** vs. **8**), while substitution at the same 3-OH position by a glycerol group positively compensate for cytotoxicity (**16** vs. **9**). In addition, compounds **1**, **6**, **9**–**11** showed selective cytotoxicity against different nonsolid cancer cell lines (U937, HL-60, and K562). Interestingly, only compounds **6**, **10** and **11**, which possess two adjacent hydroxy groups in one of the benzene rings and no substitution at C-2 position in the other ring exhibited varied inhibitory cytotoxicity on K562 cells. Moreover, by comparison of the structures of **9** and **3**–**4**, **7**, **11**–**12** and **16** having a carboxyl group, it was found that when the carboxyl group in the benzene ring is adjacent to a free hydroxy group, the cytotoxicity against HL-60 cells is lost. In summary, we found that when the ortho position of the carboxyl group in the diphenyl ethers is a free phenolic hydroxy group, it will lose all cytotoxicity against cancer cells, and the adjacent phenolic hydroxy groups confer selective cytotoxicity against several cell lines.

## 3. Materials and Methods

### 3.1. General Experimental Procedures

Optical rotations were measured with a JASCO P-2000 automatic digital polarimeter (JASCO, Easton, MD, USA). IR spectra were taken on a Nicolet 5700 FT-IR spectrometer (Termo Electron Corporation, Madison, WI, USA). The NMR spectra in CD_3_OD and DMSO-*d*_6_ with TMS as internal reference were obtained on a Bruker AVANCE III HD 600 MHz spectrometer equipped with a 5 mm cryogenic CPDCH probe (Bruker, Fällanden, Switzerland). HRESIMS were recorded on an Agilent Technologies 6520 Accurate Mass Q-TOF LC/MS spectrometer (Agilent Technologies, Santa Clara, CA, USA). Column chromatography (CC) was carried out on Sephadex LH-20 (GE Healthcare, Sweden), silica gel (300–400 mesh, Qingdao Marine Chemical Inc., Qingdao, China) and MCI gel CHP 20P/P120 (Middle Chromatogram Isolated Gel, Mitsubishi Chemical Corporation, Tokyo, Japan). LC-ESI-MS analyses were performed on a Bruker micrOTOF-Q II (Bruker, Billerica, MA, USA) using a COSMOSIL C_18_ column (5 μm, 4.6 × 250 mm). TLC was performed on GF254 plates (Qingdao Marine Chemical Factory, Qingdao, China). Medium pressure liquid chromatography (MPLC) was carried out on a TELEDYNE ISCO CombiFlash Rf+ [Universal Technology, Hong Kong, China]. HPLC was conducted using a SSI instrument with a Series 1500 photo diode array detector and COSMOSIL C_18_ column (5 μm, 4.6 × 250 mm). Standards of d/l-ribose and d/l-glucose were purchased from Sigma (St. Louis, MO, USA), and Derivatization reagents L-cysteine and o-tolyl isothiocyanate were purchased from J&K Scientific Ltd. (Beijing, China).

### 3.2. Fungal Material

The fungal strain FNA026 was isolated from marine water collected in the sea of China, Xiamen. The voucher specimen is deposited in our laboratory at −80 °C. The partial 18S rRNA sequence was compared to sequences in available databases using the Basic Local Alignment Search Tool and strain FNA026 determined to be an *Aspergillus sydowii* (Supplementary Information Figure S57).

### 3.3. Fermentation

The fungal strain FNA026 was grown on potato dextrose agar at 28 °C for 5 days. Five pieces (0.5 × 0.5 cm^2^) of mycelial agar plugs were inoculated into 500 mL Erlenmeyer flasks containing 300 mL of potato dextrose broth, which were then incubated on a rotary shaker at 250 rpm and 28 °C for 3 days. Then the seed liquid was spread in 500 mL Roux flasks (30 flasks) containing rice (100 g per flask) and artificial seawater (120 mL per flask). The flasks were incubated at 28 °C for 4 weeks.

### 3.4. Extraction and Isolation

The extraction and isolation procedures were guided by LC-MS screening with UV absorption characteristics (207 nm and 270 nm) and molecular weight (*m*/*z* 230–280 and *m*/*z* 380–480) as search criteria. The fermented rice inoculated with FNA026 (3 kg) was extracted three times with ethyl acetate (500 mL) at room temperature under sonication to give a crude extract (28.86 g), which was then dissolved in MeOH, and extracted three times using petroleum ether to afford MeOH-soluble (22.24 g) and petroleum ether-soluble (5.65 g) fractions. The MeOH-soluble fraction was subjected to MCI gel (Middle Chromatogram Isolated Gel) with a stepped gradient of MeOH–H_2_O (20:80, 50:50, 90:10, 100:0 *v*/*v*) to give 4 fractions (A–D). Fraction C (2.79 g) was separated on a silica gel column eluting with a dichloromethane-methanol gradient (1:0–0:1, *v*/*v*) to give 12 fractions (C1–C12). Fraction C4 (0.83 g) was subjected to MPLC eluting with a gradient of increasing MeCN (20–50%) in H_2_O to give 8 fractions (C4A–C4H), where pure compound **8** (328.6 mg) was obtained from fraction C4D. Fraction C4A (15.6 mg) was further purified by HPLC (1.0 mL/min; 46% MeCN in H_2_O) to give compound **9** (*t*_R_ 15.6 min, 3.5 mg). Fraction C4E (5.3 mg) was further purified by HPLC (1.0 mL/min; 64% MeOH in H_2_O) to give compound **5** (*t*_R_ 13.2 min, 1.2 mg). Fraction C4H (22.8 mg) was further purified by HPLC (1.0 mL/min; 69% MeOH in H_2_O) to give compound **13** (*t*_R_ 22.2 min, 3.3 mg). Fraction C5 (390.4 mg) was subjected to MPLC eluting with a gradient of MeCN (30–60%) in H_2_O to yield into 6 fractions (C5A–C5F). Fraction C5A (38.3 mg) was further purified by HPLC (1.0 mL/min; 59% MeOH in H_2_O) to give compound **10** (*t*_R_ 17.3 min, 5.2 mg). Fraction C6 (58.8 mg) was chromatographed over SephadexLH-20 and eluted with MeOH to yield fractions C6A–C6G. Pure compound **11** (3.7 mg) was obtained directly from fraction C6C. Fraction C7 (88.3 mg) was subjected to HPLC (1.0 mL/min; 32% MeCN in H_2_O, 0.1%TFA) to yield into 4 fractions (C7A–C7D). Pure compound **14** (3.8 mg) was obtained directly from fraction C7B. Fraction C7D (10.9 mg) was further purified by HPLC (1.0 mL/min; 68% MeOH in H_2_O) to give compound **17** (*t*_R_ 26.3 min, 3.1 mg). Fraction C9 (22.1 mg) was purified by HPLC (1.0 mL/min; 43% MeCN in H_2_O, 0.1%TFA) to give compound **15** (*t*_R_ 12.1 min, 2.2 mg). Fraction C10 (476.4 mg) was subjected to MPLC eluting with a gradient of acetonitrile (30–53%) in H_2_O to give 7 fractions (C10A–C10G), and two pure compounds **2** (5.2 mg) and **16** (4.2 mg) were obtained directly from fraction C10C and fraction C10E, respectively. Fraction C10F (165.4 mg) was applied to a Sephadex LH-20 column chromatography eluted with MeOH to give 17 fractions (C10F1–C10F17). Fraction C10F7 (8.2 mg) was further purified by HPLC (1.0 mL/min; 42% MeCN in H_2_O, 0.1%TFA) to give compound **4** (*t*_R_ 14.2 min, 1.7 mg). Fraction C10F10 (25.8 mg) was further purified by HPLC (1.0 mL/min; 44% MeCN in H_2_O) to give compound **6** (*t*_R_ 13.4 min, 1.6 mg). Fraction C11 (42.5 mg) was purified by HPLC (1.0 mL/min; 33% MeCN in H_2_O, 0.1%TFA) to give compound **12** (*t*_R_ 18.5 min, 6.4 mg). Fraction C12 (495.6 mg) was subjected to the Sephadex LH-20 column chromatography eluted with MeOH to give 8 fractions (C12A–C12H). Fraction C12G (28.6 mg) was purified by HPLC (1.0 mL/min; 34% MeCN in H_2_O, 0.1%TFA) to give compound **1** (*t*_R_ 24 min, 1.3 mg). Fraction C12H (18.6 mg) was further purified by HPLC (1.0 mL/min; 36% MeCN in H_2_O, 0.1%TFA) to give compound **3** (*t*_R_ 20 min, 2.2 mg). Fraction D (1.33 g) was subjected to column chromatography on silica gel and eluted with dichloromethane–methanol gradient (1:0–0:1, *v*/*v*), which gave 8 fractions (D1–D8). Fraction D5 (325.2 mg) was further by MPLC eluting with a gradient of increasing MeCN (50–100%) in H_2_O to give 5 fractions (D5A–D5E), and pure compound **7** (185.6 mg) was obtained directly from fraction D5C.

#### 3.4.1. Cordyol C-3-*O*-*α*-d-ribofuranoside (**1**)

Colorless oil; [α]${}_{D}^{25}$ −8.0 (*c* 0.20, MeOH), UV(MeOH) *λ*_max_ (log *ε*): 204.8 (4.02) nm, 279.4 (3.25) nm; IR *ν*_max_: 3320.1, 1677.7, 1596.2, 1512.1, 1464.3, 1322.1, 1210.8, 1140.0, 1046.8, 997.6, 836.2 cm^−1^; ^1^H and ^13^C NMR spectroscopic data see Table 1 and Table 2; HRESIMS *m*/*z* 401.1206 [M + Na]^+^ (calcd. for C_19_H_22_O_8_Na, 401.1207).

#### 3.4.2. Diorcinol-3-*O*-*α*-d-ribofuranoside (**2**)

Colorless oil; [α]${}_{D}^{25}$ −18.6 (*c* 0.40, MeOH), UV(MeOH) *λ*_max_ (log *ε*): 207.2 (4.05) nm, 273.4 (3.25) nm; IR *ν*_max_: 3344.2, 2931.4, 1600.7, 1464.7, 1325.1, 1154.9, 1039.8, 839.2 cm^−1^; ^1^H and ^13^C NMR spectroscopic data see Table 1 and Table 2; HRESIMS *m*/*z* 385.1261 [M + Na]^+^ (calcd. for C_19_H_22_O_7_Na, 385.1258).

#### 3.4.3. 4-Methoxycarbonyl Diorcinol-3-*O*-*α*-d-glucoside (**3**)

Colorless oil; [α]${}_{D}^{25}$ +9.6 (*c* 0.20, MeOH), UV(MeOH) *λ*_max_ (log *ε*): 214.2 (4.03) nm, 261.5 (3.64) nm, 298.4 (3.28) nm; IR *ν*_max_: 3334.5, 1651.3, 1579.3, 1454.2, 1324.9, 1268.4, 1161.2, 1023.9, 847.5 cm^−1^; ^1^H and ^13^C NMR spectroscopic data see Table 1 and Table 2; HRESIMS *m*/*z* 473.1403 [M + Na]^+^ (calcd. for C_22_H_26_O_10_Na, 473.1418).

#### 3.4.4. 2-(Ethoxycarbonyl)-4′-carboxydiorcinal (**4**)

Colorless oil; UV(MeOH) *λ*_max_ (log *ε*): 216.6 (4.04) nm, 259.2 (3.59) nm, 299.6 (3.26) nm; IR *ν*_max_: 3251.1, 1654.4, 1613.0, 1460.1, 1317.8, 1260.4, 1167.0, 845.9, 802.3 cm^−1^; ^1^H and ^13^C NMR spectroscopic data see Table 1 and Table 2; HRESIMS *m*/*z* 369.0954 [M + Na]^+^ (calcd. for C_18_H_18_O_7_Na, 369.0945).

#### 3.4.5. 7-Ethyldiorcinol (**5**)

Colorless oil; UV(MeOH) *λ*_max_ (log *ε*): 207.2 (4.03) nm, 280.5 (3.27) nm; IR *ν*_max_: 3343.5, 1598.3, 1459.8, 1329.8, 1155.3, 995.4, 841.3 cm^−1^; ^1^H and ^13^C NMR spectroscopic data see Table 1 and Table 2; HRESIMS *m*/*z* 245.1183 [M + H]^+^ (calcd. for C_15_H_17_O_3_, 245.1172).

#### 3.4.6. 3-Hydroxydiorcinol (**6**)

Colorless oil; UV(MeOH) *λ*_max_ (log *ε*): 221.3 (4.03) nm, 280.5 (3.71) nm; IR *ν*_max_: 3286.7, 1598.2, 1491.4, 1324.2, 1154.0, 1024.7, 976.7, 836.8 cm^−1^; ^1^H and ^13^C NMR spectroscopic data see Table 1 and Table 2; HRESIMS *m*/*z* 245.0808 [M − H]^−^ (calcd. for C_14_H_13_O_4_, 245.0819).

### 3.5. Determination of the Absolute Configuration of Sugar Moieties in ***1**–**3***

To determine the absolute configurations of sugar moieties in **1**–**3**, a modified method based on LC-ESI-MS analysis was performed, where the retention time of sugar samples obtained after hydrolysis of the parent compounds were compared with those from standard sugars (d/l) [34]. In detail, compounds **1**, **2** and **3** (approximately 0.05 mg, each) were hydrolyzed with 2 mol/L HCl (400 μL) in a 2 mL glass vial at 80 °C for 4 h. The reaction mixture was then diluted with H_2_O (400 μL) and extracted with CHCl_3_ (400 μL) three times. The aqueous layer containing monosaccharides was concentrated *in vacuo* to yield a dried sugar mixture. The resulting sugar mixture (not weighed out) and standard sugar samples (d/l-ribose and d/l-glucose, 0.1 mg for each) respectively, were heated with l-cysteine methyl ester (0.1 mg) in pyridine (400 μL) in a 2 mL glass vial at 60 °C for 60 min, then o-tolyl isothiocyanate (200 μL) was added to the reaction mixture and kept at 60 °C for additional 60 min. Then, the reaction mixture was directly analyzed by LC-ESI-MS (COSMOSIL 5 μm, 4.6 × 250 mm, C_18_ column). Analysis was performed at 30 °C with a flow rate of 1.0 mL/min, and the elution was carried out using a gradient of MeCN (0–30 min, 10–50%, linear gradient) in H_2_O. Source parameters in the positive ion mode were set as follows: Capillary entrance voltage = −4500 V, end plate offset = −500 V, nebulizer pressure (N_2_) = 11.6 psi, dry gas (N_2_) = 6.0 L/min, dry gas temperature = 220 °C. High-purity nitrogen (N_2_) were used as the nebulizing gas. Ion Peaks were extracted at *m*/*z* 447 for ribose and at *m*/*z* 471 for glucose identified by comparison of retention time with those of standards. The retention time of d-ribose and d-glucose derivatives were 22.3 and 21.0 min, respectively.

### 3.6. Cytotoxicity Assay

Cancer cell lines, including 4T1, U937, PC3, HL-60, HT-29, A549, NCI-H460, and K562, were purchased from ATCC. All the cells were maintained in RPMI1640 supplemented with 10% FBS, 100 units/mL Penicillin G and 100 μg/mL streptomycin. All the cancer cells were incubated at 37 °C in humidified air containing 5% CO_2_. MTT assay was used to determine the cell viability. Cells were seeded in 96-well plates at 1.5–3.0 × 10^4^/mL (100 μL/well). After 24 h incubation, 5 different concentrations (final concentrations were 1.6, 3.1, 6.3, 12.5 and 25.0 μM) of tested compounds were added into the wells in triplicate. Five concentrations of doxorubicin were tested, including 2.0, 1.0, 0.5, 0.25, and 0.125 μM. Cells were incubated for 96 h before MTT was added into the cells at a final concentration of 500 μg/mL, and the plates were incubated for an additional 4 h. The resultant formazan crystals were dissolved in 200 μL of DMSO, then a microplate reader (Synergy HT, Bio-Tek) was used to measure the absorbance of the plates at 570 nm for testing the cell viability of serial concentrations of compounds and the IC_50_ were estimated.

## 4. Conclusions

A total of 17 diphenyl ethers, including 6 new compounds, were isolated from a marine-derived *Aspergillus sydowii.* Compounds **1** and **2** are rare diphenyl ether glycosides containing a d-ribofuranose moiety. Although natural diphenyl ethers have been extensively investigated, their structures still exhibit variability due to the presence of hydroxy groups, and the diversity and location of sugar moieties. Furthermore, a modified method based on LC-MS analysis was used to determine the absolute configuration of sugar moieties. Comparing to conventional method based on LC-UV, which normally requires 0.5–3 mg of sample [34,35,36,37], our method has higher sensitivity due to the use of ESIMS detection, and as low as 0.05 mg of sample was enough to determine the absolute configuration of the sugar units using this procedure. Moreover, all the compounds were evaluated for their cytotoxicity against eight cancer cell lines, and compounds **1**, **5**, **6**, and **8**–**11** were found to exhibit highly selective cytotoxicities against different cancer cell lines.

## Figures and Tables

**Table 1:** The ^1^H NMR spectroscopic data for compounds **1**–**6** and **6a** (600 MHz).

NO.	*δ*_H_ (*J* in Hz)						
	1 ^a^	2 ^b^	3 ^a^	4 ^b^	5 ^b^	6 ^b^	6a ^b^
2		6.20 t (2.2)	6.57 t (2.4)	6.24 d (2.3)	6.21 brs	6.13 t (2.4)	6.30 d (2.2)
4	6.81 brs	6.27 brs	6.75 brs	6.35 d (2.3)	6.30 m	6.28 brs	6.48 brs
6	6.46 brs	6.37 brs	6.53 brs		6.39 m	6.20 brs	6.43 brs
7	2.18 s	2.27 s	2.26 s	2.47 s	2.53 q (7.5)	2.19 s	2.26 s
8					1.18 t (7.5)		3.73 s
9				4.40 q (7.5)			
10				1.40 t (7.5)			
2′	6.02 brs	6.57 t (2.8)	6.28 d (2.3)	6.28 d (2.7)	6.21 brs	6.33 d (2.8)	6.52 d (2.8)
4′	6.21 brs	6.45 brs			6.28 brs		
6′	6.12 brs	6.72 brs	6.37 d (2.3)	6.56 d (2.7)	6.36 brs	6.26 d (2.8)	6.37 d (2.8)
7′	2.15 s	2.22 s	2.21 s	2.35 s	2.22 s	2.17 s	2.20 s
8′							3.78 s
9′			3.78 s				3.75 s
1″	5.50 d (4.6)	5.57 d (4.5)	5.35 d (3.6)				
2″	4.07 ddd (10.7, 6.8, 4.1)	4.15 dd (6.5, 4.5)	3.33 m				
3″	3.93 ddd (11.7, 5.9, 2.7)	4.06 dd (6.5, 3.2)	3.58 t (9.2)				
4″	3.98 q (4.0)	4.12 dd (6.9, 3.5)	3.17 t (9.2)				
5″	3.46 brt (5.1)	3.63 dd (12.1, 3.9) 3.69 dd (11.7, 3.4)	3.42 m				
6″			3.47 dd (11.7, 5.2)				
			3.55 dd (11.8, 1.8)				
2′-OH	8.41 s						
3″-OH	9.26 s		10.26 s				
2′-OH	5.14 d (6.0)						
3″-OH	5.16 brs						
5″-OH	4.81 t (5.6)						

^a^ Recorded in DMSO-*d*_6_; ^b^ Recorded in CD_3_OD.

**Table 2:** ^13^C NMR spectroscopic data for compounds **1**–**6** and **6a** (150 MHz).

NO.	*δ*_C_, Type						
	1 ^a^	2 ^b^	3 ^a^	4 ^b^	5 ^b^	6 ^b^	6a ^b^
1	142.0 (C)	159.6 (C)	156.1 (C)	163.2 (C)	159.5 (C)	161.2 (C)	160.2 (C)
2	137.3 (C)	106.3 (CH)	105.5 (CH)	103.9 (C)	104.5 (CH)	102.8 (CH)	102.6 (CH)
3	145.7 (C)	159.5 (C)	158.3 (C)	164.9 (C)	159.7 (C)	159.4 (C)	162.3 (C)
4	112.9 (CH)	111.8 (CH)	113.5 (CH)	113.3 (CH)	111.7 (CH)	111.0 (CH)	110.3 (CH)
5	127.3 (C)	141.6 (C)	140.5 (C)	144.1 (C)	148.2 (C)	141.4 (C)	141.7 (C)
6	115.9 (CH)	112.1 (CH)	113.6 (CH)	109.7 (CH)	110.8 (CH)	110.4 (CH)	112.2 (CH)
7	20.6 (CH_3_)	21.6 (CH_3_)	21.1 (CH_3_)	23.9 (CH_3_)	29.8 (CH_2_)	21.6 (CH_3_)	21.7 (CH_3_)
8				172.4 (C)	15.9 (CH_3_)		55.7 (CH_3_)
9				62.5 (CH_2_)			
10				14.5 (CH_3_)			
1′	159.1 (C)	159.7 (C)	158.9 (C)	160.8 (C)	159.6 (C)	150.1 (C)	154.3 (C)
2′	100.3 (CH)	104.3 (CH)	102.6 (CH)	106.4 (CH)	104.2 (CH)	106.1 (CH)	103.4 (CH)
3′	158.2 (C)	159.4 (C)	157.6 (C)	155.0 (C)	159.7 (C)	146.8 (C)	154.8 (C)
4′	109.6 (CH)	114.2 (CH)	114.9 (C)	120.1(C)	111.9 (CH)	140.8 (C)	144.5 (C)
5′	139.5 (C)	141.7 (C)	139.1 (C)	140.6 (C)	141.6 (C)	126.7 (C)	133.5 (C)
6′	107.5 (CH)	113.5 (CH)	110.7 (CH)	114.9 (CH)	110.6 (CH)	113.6 (CH)	113.7 (CH)
7′	21.2 (CH_3_)	21.5 (CH_3_)	20.1 (CH_3_)	20.2 (CH_3_)	21.5 (CH_3_)	16.2 (CH_3_)	16.0 (CH_3_)
8′			168.5 (C)	170.7 (C)			56.3 (CH_3_)
9′			51.9 (CH_3_)				60.6 (CH_3_)
1″	101.3 (CH)	102.3 (CH)	97.9 (CH)				
2″	72.3 (CH)	73.4 (CH)	71.5 (CH)				
3″	69.4 (CH)	71.2 (CH)	73.0 (CH)				
4″	86.7 (CH)	87.5 (CH)	69.8 (CH)				
5″	61.6 (CH_2_)	63.2 (CH_2_)	73.8 (CH)				
6″			60.6 (CH_2_)				

^a^ Recorded in DMSO-*d*_6_; ^b^ Recorded in CD_3_OD.

**Table 3:** Cytotoxicity (IC_50_ in µM) for compounds **1**, **5**–**6**, **8**–**11**.

Compounds	A549	U937	HL-60	K562
**1**	8.97 ± 0.48	4.64 ± 0.35	/	/
**5**	16.13 ± 1.24	/	/	/
**6**	/	/	11.98 ± 0.73	18.89 ± 1.14
**8**	15.51 ± 1.59	/	/	/
**9**	3.36 ± 0.68	/	21.22 ± 1.25	/
**10**	/	/	16.52 ± 0.99	20.88 ± 1.60
**11**	/	/	13.33 ± 0.87	23.03 ± 1.34
**DOX**	0.19 ± 0.04	<0.125	<0.125	0.49 ± 0.08

“/” no cytotoxicity was detected. Inactive compounds were not shown here.

## Supplementary Materials

The following are available online at http://www.mdpi.com/1660-3397/16/11/451/s1, Figures S1–S53: HRESIMS, 1D and 2D NMR, IR, and UV spectra of **1**–**19** and **6a**, Figures S54–S56: LC-ESI-MS analysis of sugar derivatives of **1**–**3**, **Figure S57**: the internal transcribed spacers (ITS) sequence of strain FNA026.
