# Supplementary material for: Diphenyl Ethers from a Marine-Derived Aspergillus sydowii
Source: Mar Drugs. 2018 Nov 16;16(11):451. doi: 10.3390/md16110451 (PMC6267227; doi:10.3390/md16110451)
Supplement: Supplementary file 1 [file marinedrugs-16-00451-s001.pdf]

Diphenyl Ethers from a Marine-Derived *Aspergillus sydowii*

Ya-Nan Wang [2,†], Yan-Hua Mou [1,†], Yu Dong [1,2,†], Yan Wu [2], Bing-Yu Liu [2], Jian Bai [2], Dao-Jiang Yan [2], Le Zhang [2], Dan-Qing Feng [3], Yue-Hu Pei [1,*] and You-Cai Hu [2,*]

[1] Shenyang Pharmaceutical University, Shenyang 110016, China; mu_hua_jj@sina.com (Y.-H.M.); allenn7@foxmail.com (Y.D.);

[2] State Key Laboratory of Bioactive Substance and Function of Natural Medicines, Institute of Materia Medica, Chinese Academy of Medical Sciences and Peking Union Medical College, Beijing 100050, China; wangyanan@imm.ac.cn (Y.-N.W.); wuyan@imm.ac.cn (Y.W.); liubingyu@imm.ac.cn (B.-Y.L.); baijian@imm.ac.cn (B.J.); yandj@imm.ac.cn (Y.-D.J.); zhangle@imm.ac.cn (L. Z.)

[3] State-Province Joint Engineering Laboratory of Marine Bioproducts and Technology, College of Ocean & Earth Sciences, Xiamen University, Xiamen, 361102, China; dqfeng@xmu.edu.cn (D.-Q.F.)

* Correspondence: peiyueh@vip.163.com (Y.-H.P.); huyoucai@imm.ac.cn (Y.-C.H.); Tel.: +86-024-23986485 (Y.-H.P.); +86-010- 61271883 (Y.-C.H.)

† These authors contributed equally to this paper.

The List of Contents

No.	Content	Page
1	**Figure S1**. IR spectrum of compound **1**	4
2	**Figure S2**. UV spectrum of compound **1**	4
3	**Figure S3**. ^{1}H NMR spectrum of compound **1** in DMSO-*d6*	5
4	**Figure S4**. ^{13}C NMR spectrum of compound **1** in DMSO-*d6*	5
5	**Figure S5**. HMBC spectrum of compound **1** in DMSO-*d6*	6
6	**Figure S6**. HSQC spectrum of compound **1** in DMSO-*d6*	6
7	**Figure S7**. COSY spectrum of compound **1** in DMSO-*d6*	7
8	**Figure S8**. HRESIMS of compound **1**	7
9	**Figure S9**. IR spectrum of compound **2**	8
10	**Figure S10**. UV spectrum of compound **2**	8
11	**Figure S11**. ^{1}H NMR spectrum of compound **2** in CD_3OD	9
12	**Figure S12**. ^{13}C NMR spectrum of compound **2** in CD_3OD	9
13	**Figure S13**. HMBC spectrum of compound **2** in CD_3OD	10
14	**Figure S14**. HSQC spectrum of compound **2** in CD_3OD	10
15	**Figure S15**. COSY spectrum of compound **2** in CD_3OD	11
16	**Figure S16**. HRESIMS of compound **2**	11
17	**Figure S17**. IR spectrum of compound **3**	12
18	**Figure S18**. UV spectrum of compound **3**	12
19	**Figure S19**. ^{1}H NMR spectrum of compound **3** in DMSO-*d6*	13
20	**Figure S20**. ^{13}C NMR spectrum of compound **3** in DMSO-*d6*	13
21	**Figure S21**. HMBC spectrum of compound **3** in DMSO-*d6*	14
22	**Figure S22**. HSQC spectrum of compound **3** in DMSO-*d6*	14
23	**Figure S23**. J Resolved HSQC spectrum of compound **3** in CD_3OD	15
23	**Figure S24**. COSY spectrum of compound **3** in DMSO-*d6*	15
24	**Figure S25**. HRESIMS of compound **3**	15
25	**Figure S26**. IR spectrum of compound **4**	16
26	**Figure S27**. UV spectrum of compound **4**	16
27	**Figure S28** ^{1}H NMR spectrum of compound **4** in CD_3OD	17
28	**Figure S29**. ^{13}C NMR spectrum of compound **4** in CD_3OD	17
29	**Figure S30**. HMBC spectrum of compound **4** in CD_3OD	18
30	**Figure S31**. HSQC spectrum of compound **4** in CD_3OD	18
31	**Figure S32**. COSY spectrum of compound **4** in CD_3OD	19
32	**Figure S33**. HRESIMS of compound **4**	19
33	**Figure S34**. IR spectrum of compound **5**	20
34	**Figure S35**. UV spectrum of compound **5**	20
35	**Figure S36**. ^{1}H NMR spectrum of compound **5** in CD_3OD	21
36	**Figure S37**. ^{13}C NMR spectrum of compound **5** in CD_3OD	21
37	**Figure S38**. HMBC spectrum of compound **5** in CD_3OD	22
38	**Figure S39**. HSQC spectrum of compound **5** in CD_3OD	22
39	**Figure S40**. COSY spectrum of compound **5** in CD_3OD	23
40	**Figure S41**. HRESIMS of compound **5**	23

Figure S42. IR spectrum of compound **6** 24
Figure S43. UV spectrum of compound **6** 24
Figure S44. ^{1}H NMR spectrum of compound **6** in CD_3OD 25
Figure S45. ^{13}C NMR spectrum of compound **6** in CD_3OD 25
Figure S46. HMBC spectrum of compound **6** in CD_3OD 26
Figure S47. HSQC spectrum of compound **6** in CD_3OD 26
Figure S48. COSY spectrum of compound **6** in CD_3OD 27
Figure S49. HRESIMS of compound **6** 27
Figure S50. ^{1}H NMR spectrum of compound **6a** in CD_3OD 28
Figure S51. ^{13}C NMR spectrum of compound **6a** in CD_3OD 28
Figure S52. HMBC spectrum of compound **6a** in CD_3OD 29
Figure S53. HSQC spectrum of compound **6a** in CD_3OD 29
Figure S54. LC-ESI-MS analysis of derivative of sugar moiety in **1** 30
Figure S55. LC-ESI-MS analysis of derivative of sugar moiety in **2** 30
Figure S56. LC-ESI-MS analysis of derivative of sugar moiety in **3** 30
Figure S57. The internal transcribed spacers (ITS) sequence of strain FNA026 31

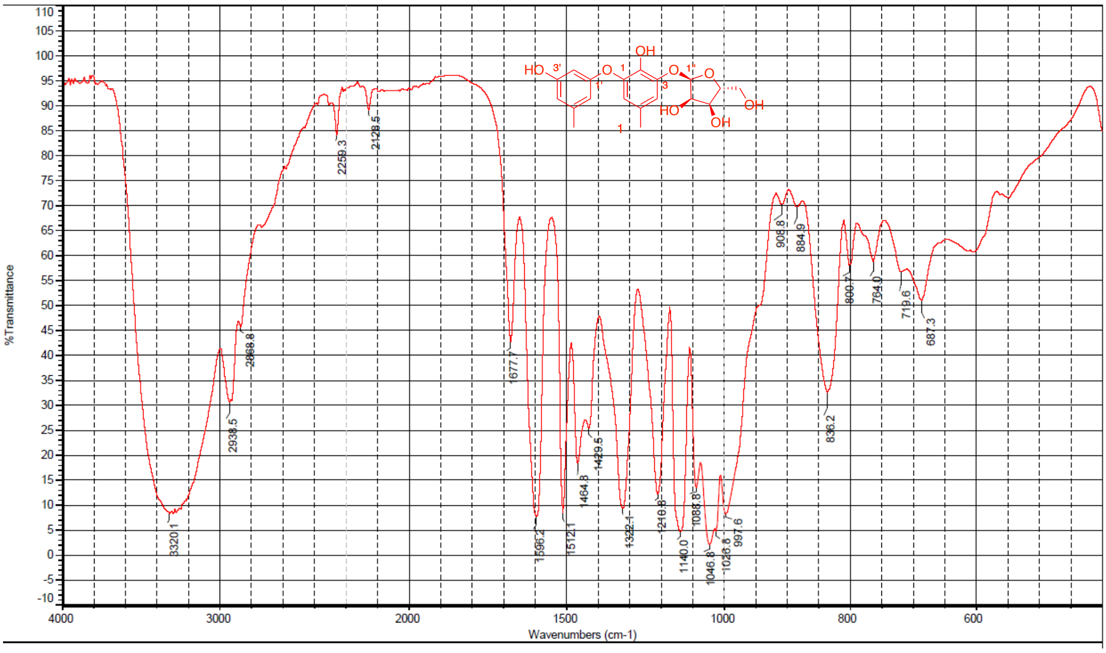

Figure S1. IR spectrum of compound **1**

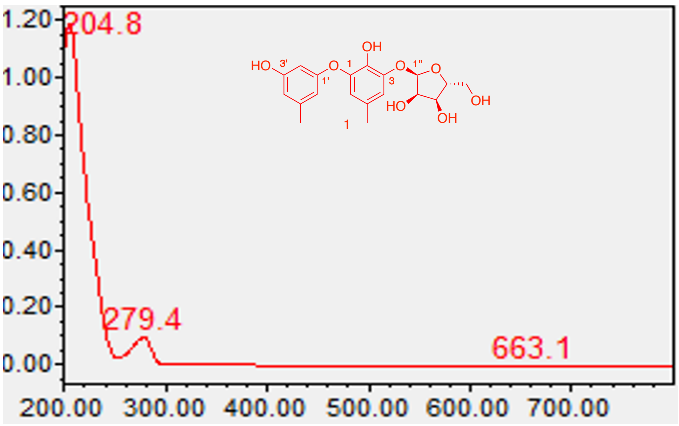

Figure S2. UV spectrum of compound **1**

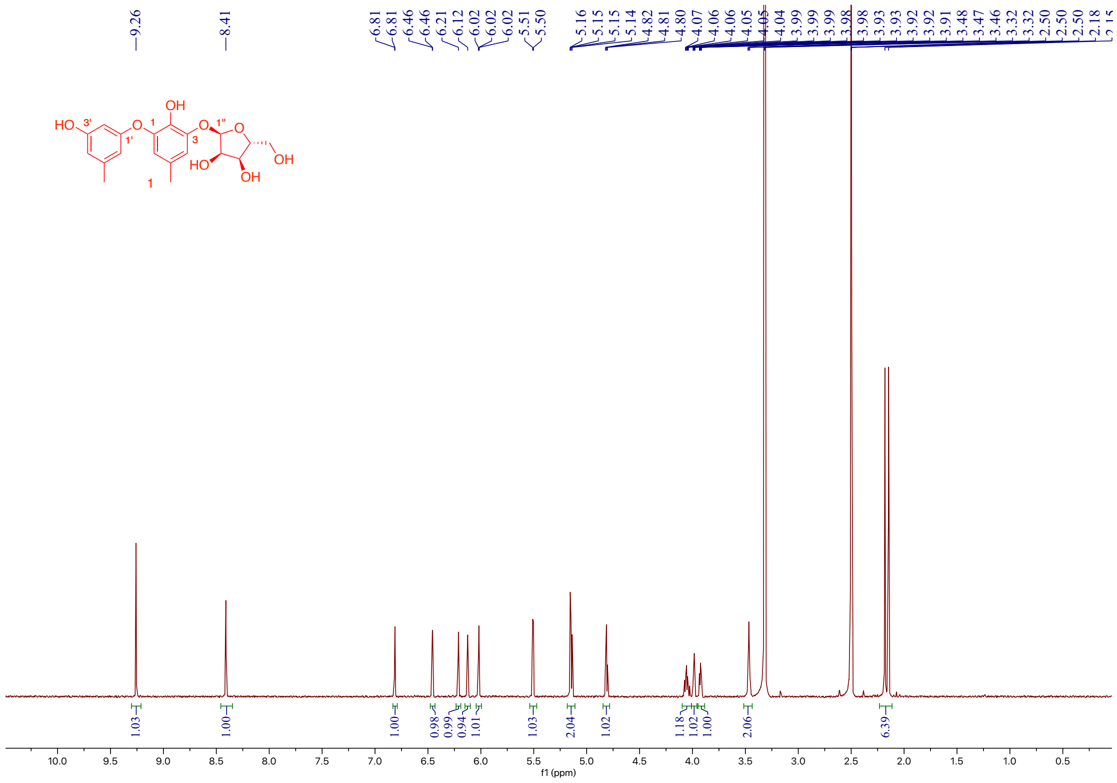

Figure S3. ^{1}H NMR spectrum of compound **1** in DMSO-d_6 (600 MHz)

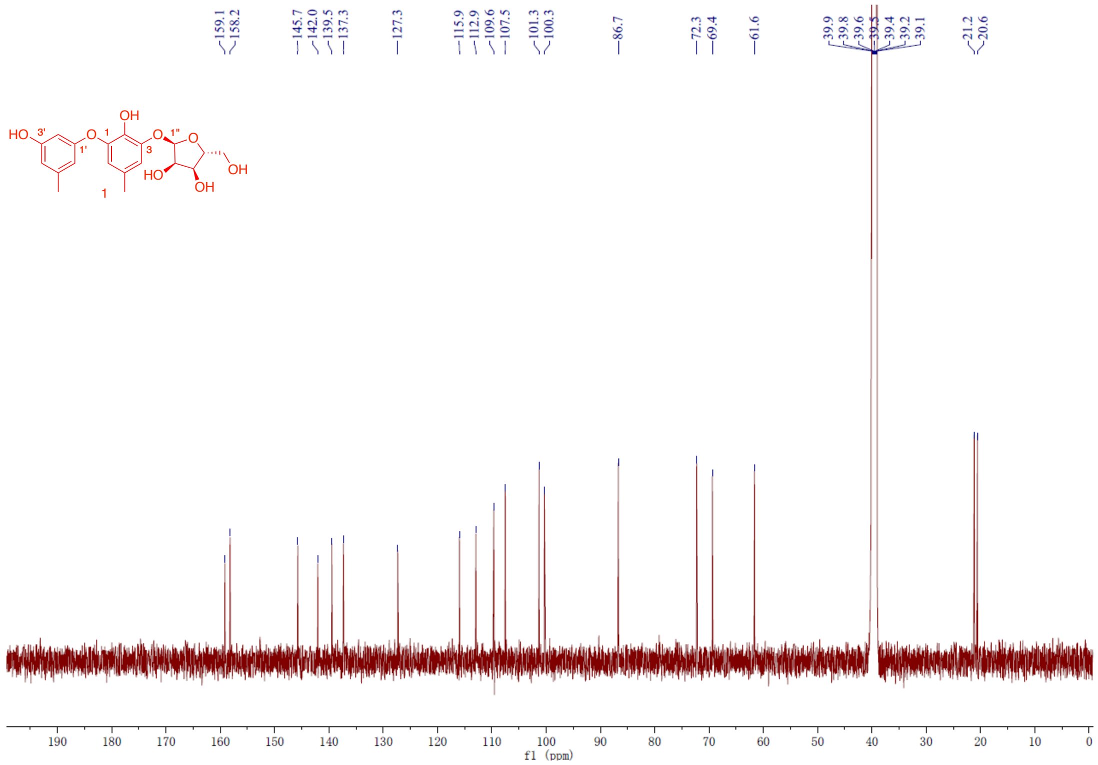

Figure S4. ^{13}C NMR spectrum of compound **1** in DMSO-d_6 (150 MHz)

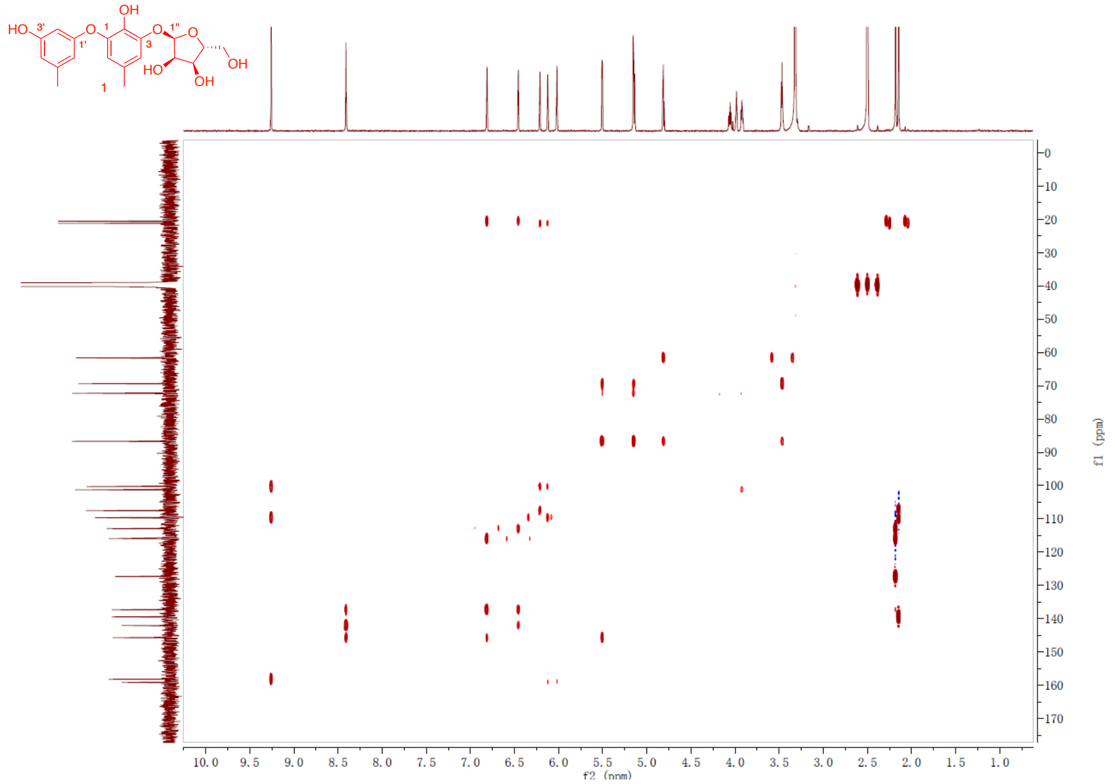

Figure S5. HMBC spectrum of compound **1** in DMSO-*d*6

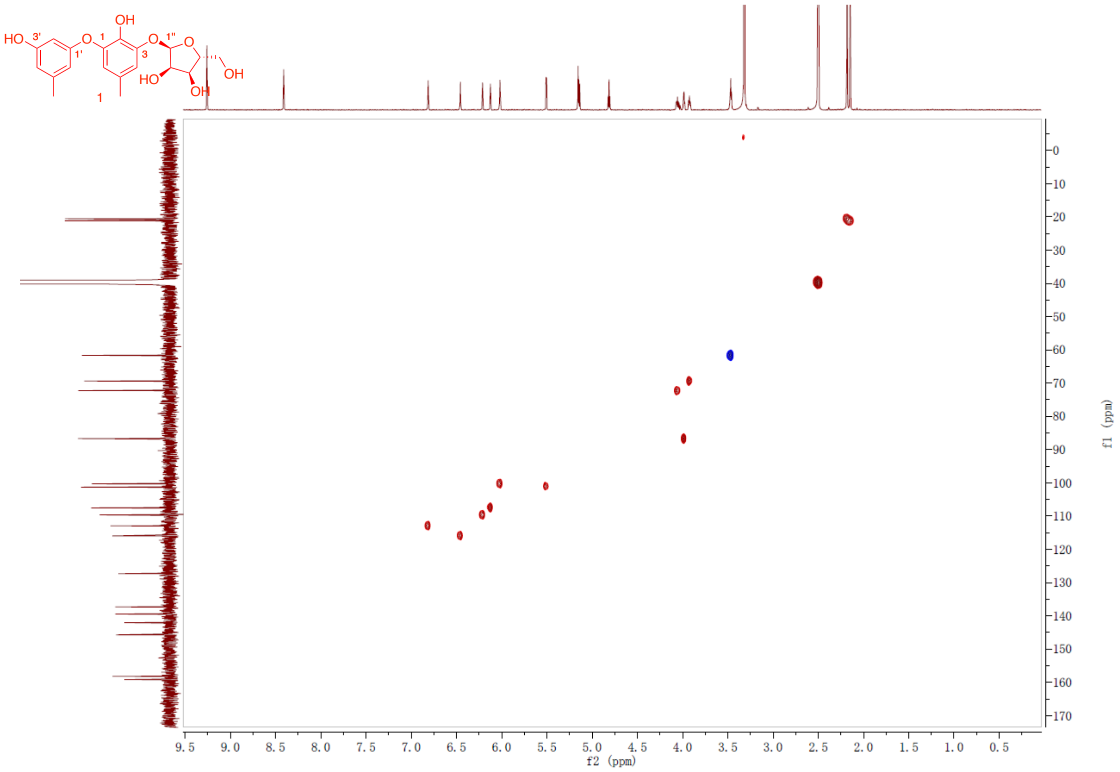

Figure S6. HSQC spectrum of compound **1** in DMSO-*d*6

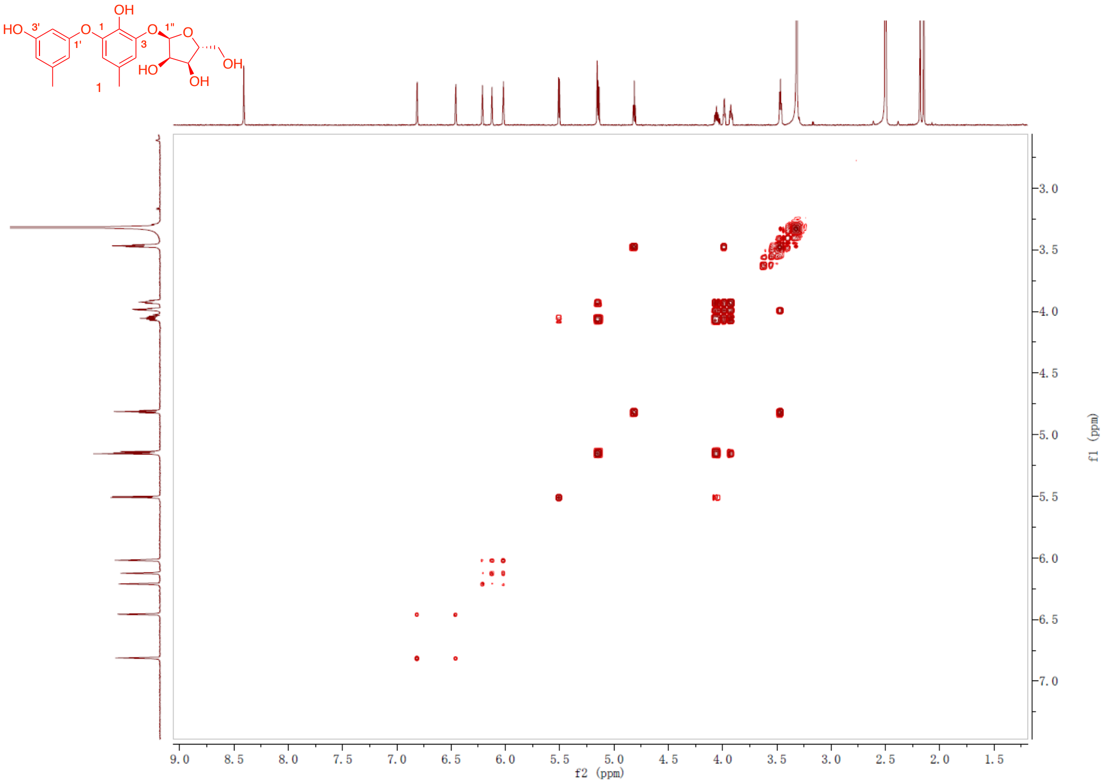

Figure S7. COSY spectrum of compound **1** in DMSO-d_6

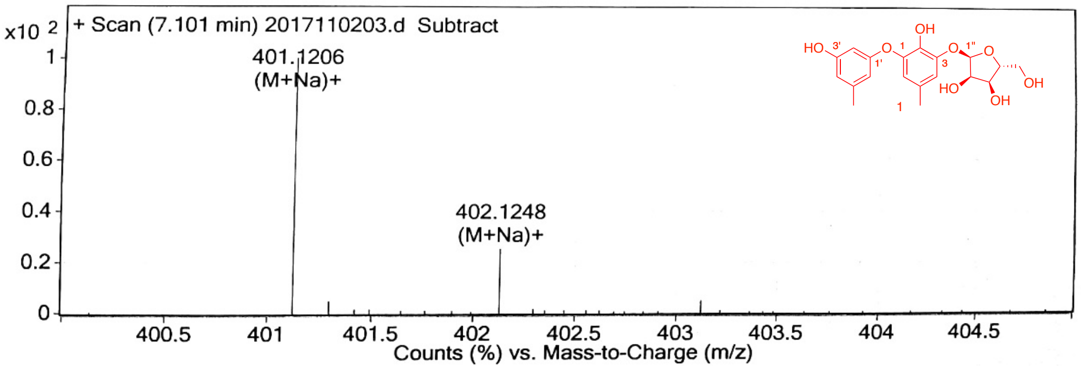

m/z	Ion	Formula	Abundance
401.1206	(M+Na)+	C19 H22 Na O8	73989.2

Best	Formula (M)	Ion Formula	Score	Cross Sco	Mass	Calc Mass	Calc m/z	Diff (ppm)	Abs Diff (ppm)	Mass Match	Abund Match	Spacing Match	DBE
☑	C19 H22 O8	C19 H22 Na O8	99.43		378.1314	378.1315	401.1207	0.09	0.09	100	98.11	99.88	9

Figure S8. HRESIMS of compound **1**

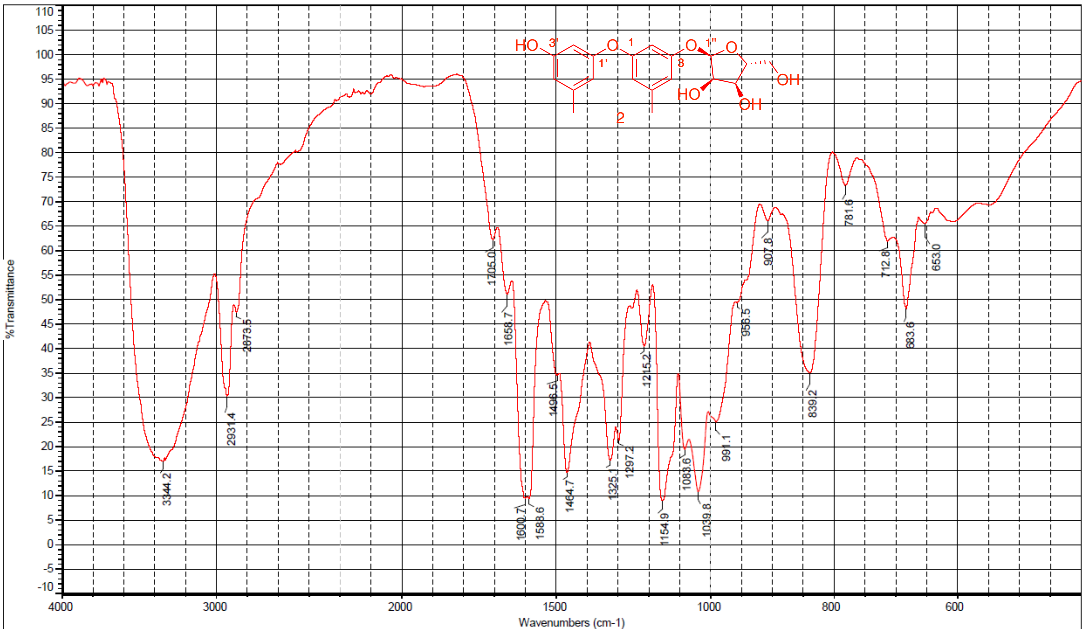

Figure S9. IR spectrum of compound **2**

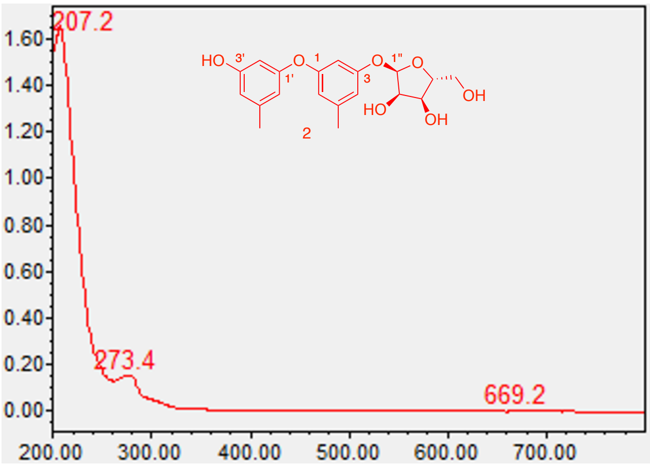

Figure S10. UV spectrum of compound **2**

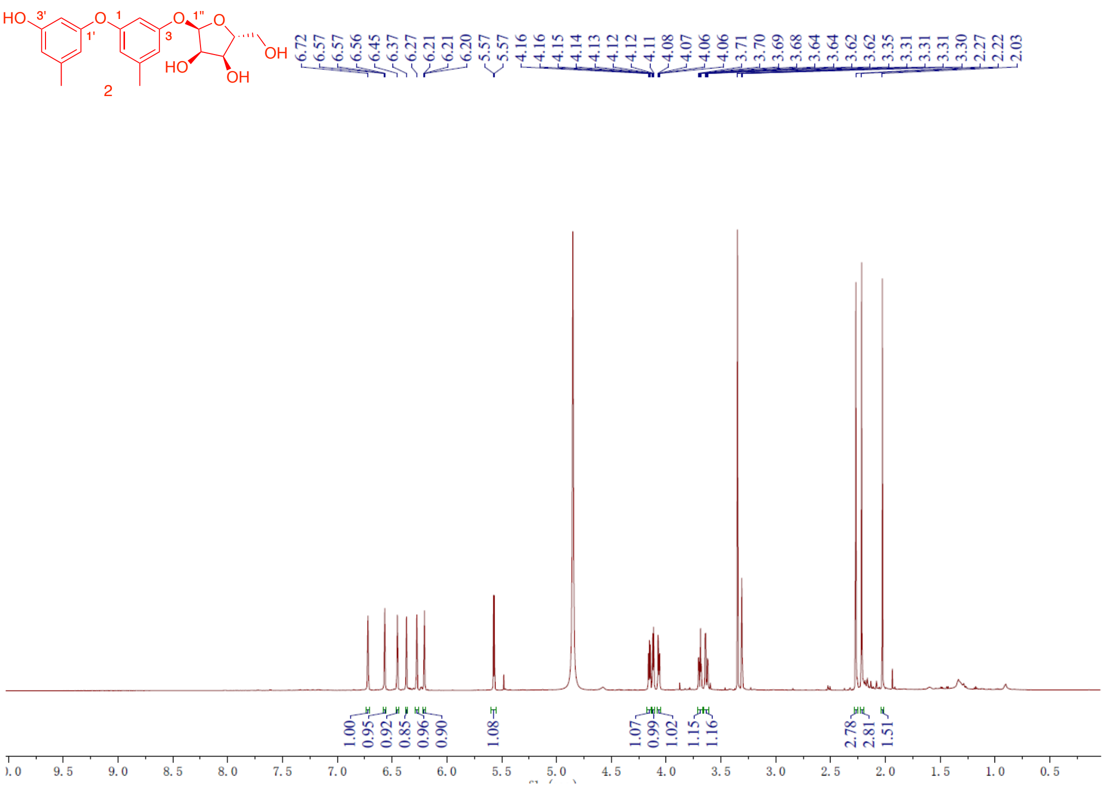

Figure S11. ^{1}H NMR spectrum of compound **2** in CD_3OD (600 MHz)

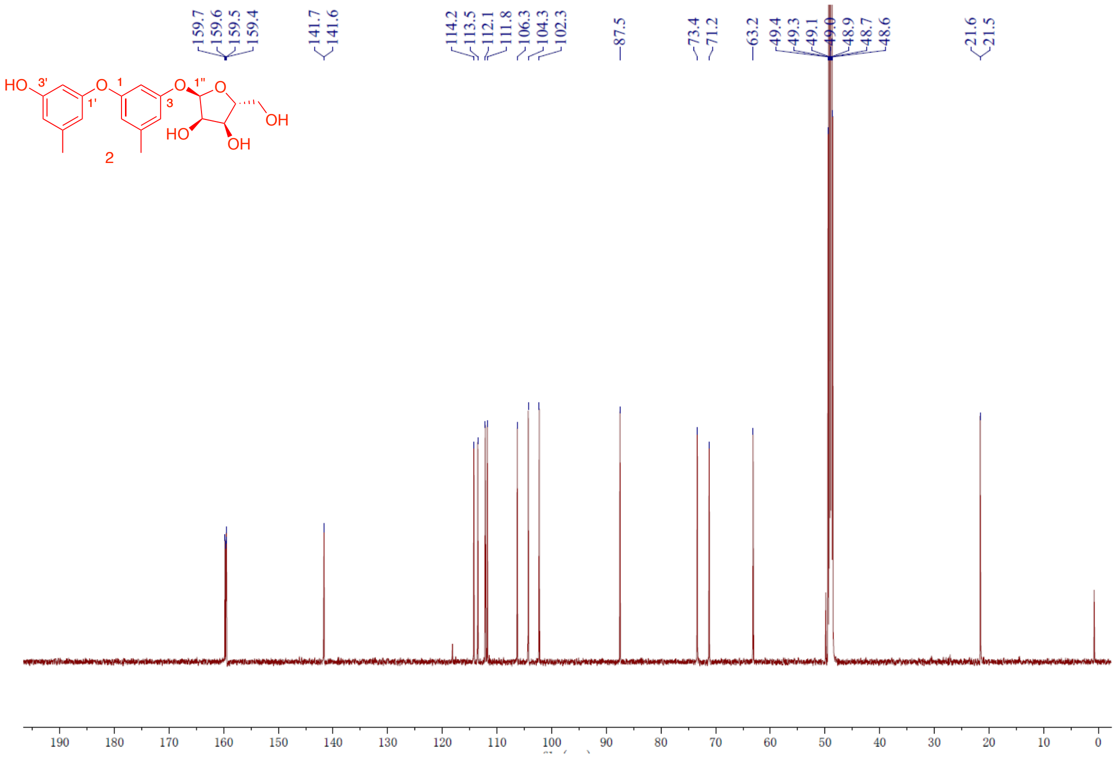

Figure S12. ^{13}C NMR spectrum of compound **2** in CD_3OD (150 MHz)

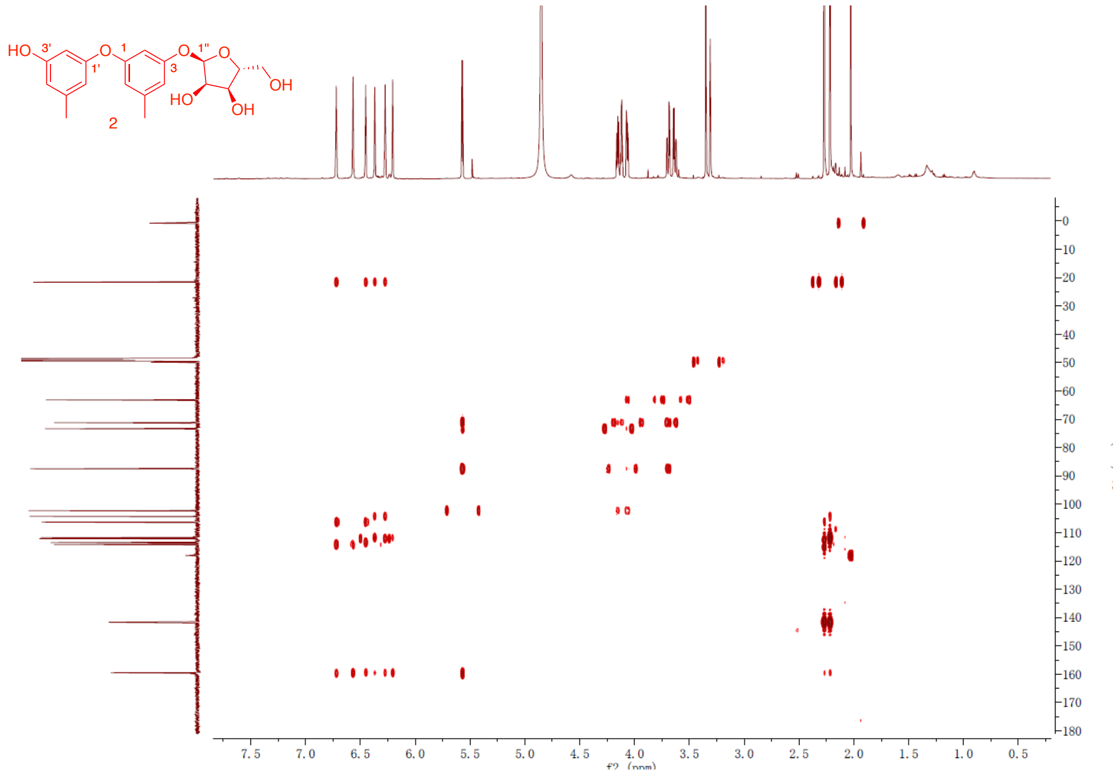

Figure S13. HMBC spectrum of compound **2** in CD3OD

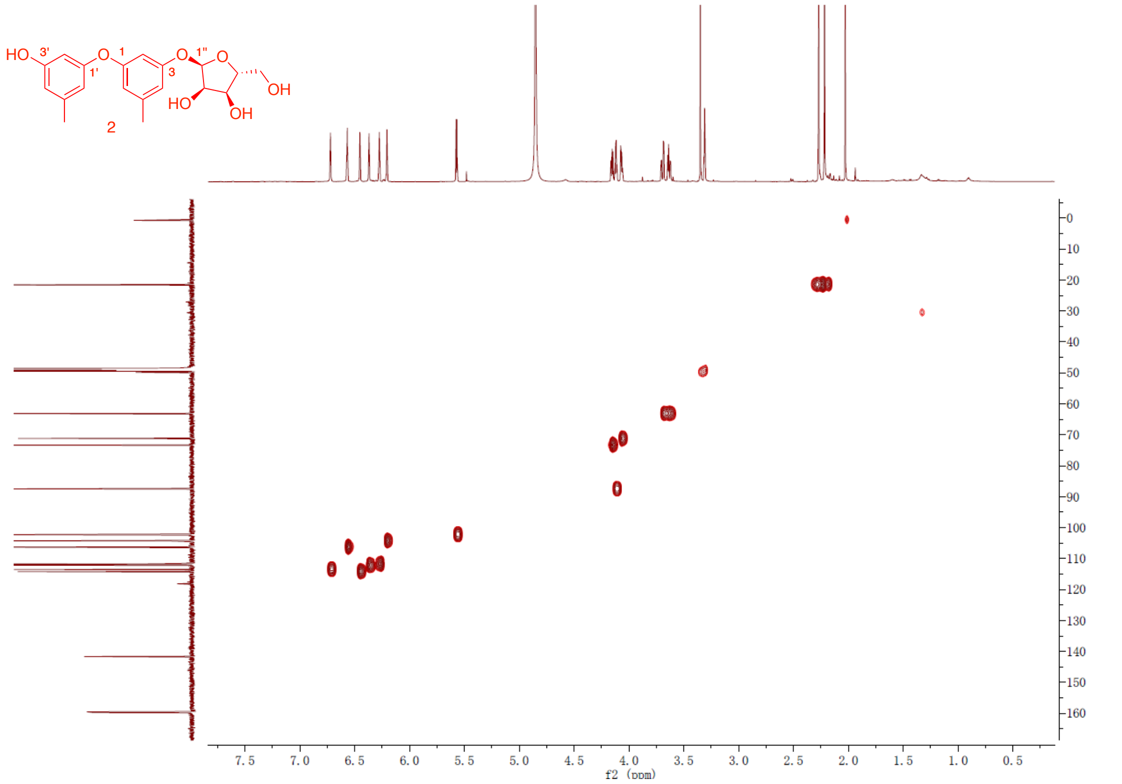

Figure S14. HSQC spectrum of compound **2** in CD3OD

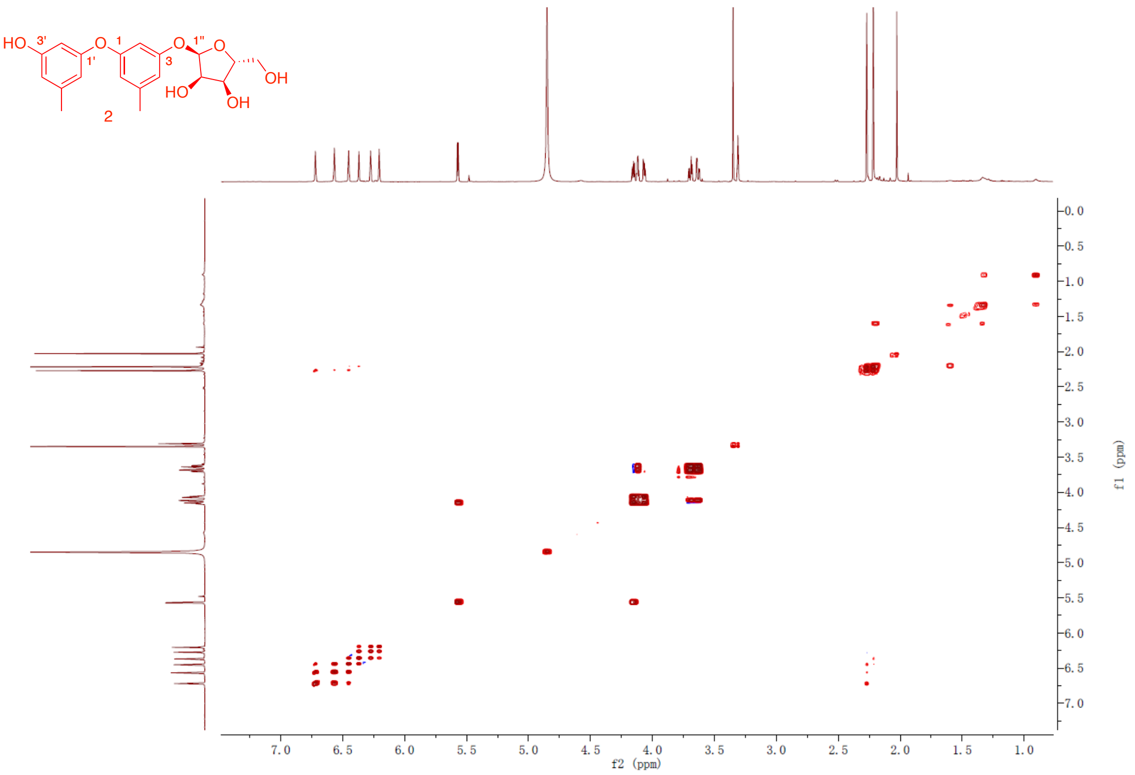

Figure S15. COSY spectrum of compound **2** in CD3OD

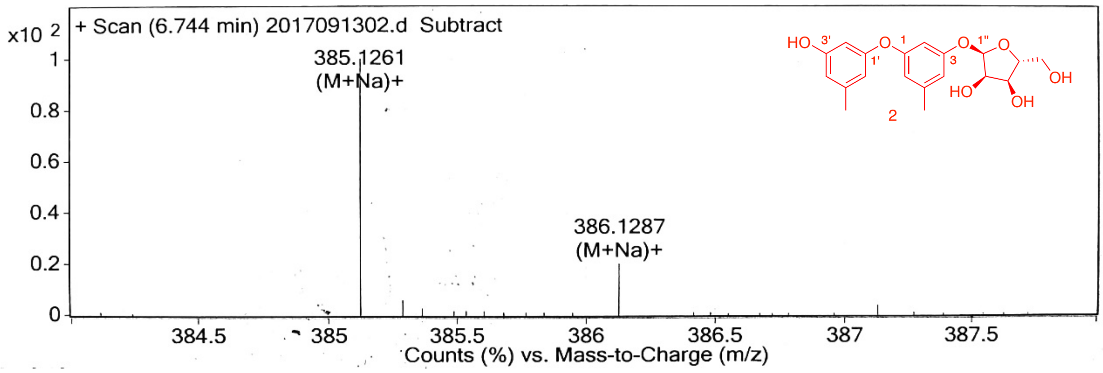

m/z	Ion	Formula	Abundance
385.1261	(M+Na)+	C19 H22 Na O7	580394.8

Best	Formula (M)	Ion Formula	Score	Cross Sco	Mass	Calc Mass	Calc m/z	Diff (ppm)	Abs Diff (ppm)	Mass Match	Abund Match	Spacing Match	DBE
✓	C19 H22 O7	C19 H22 Na O7	99.9		362.1369	362.1366	385.1258	-0.85	0.85	99.98	99.8	99.85	[illegible]

Figure S8. HRESIMS of compound **2**

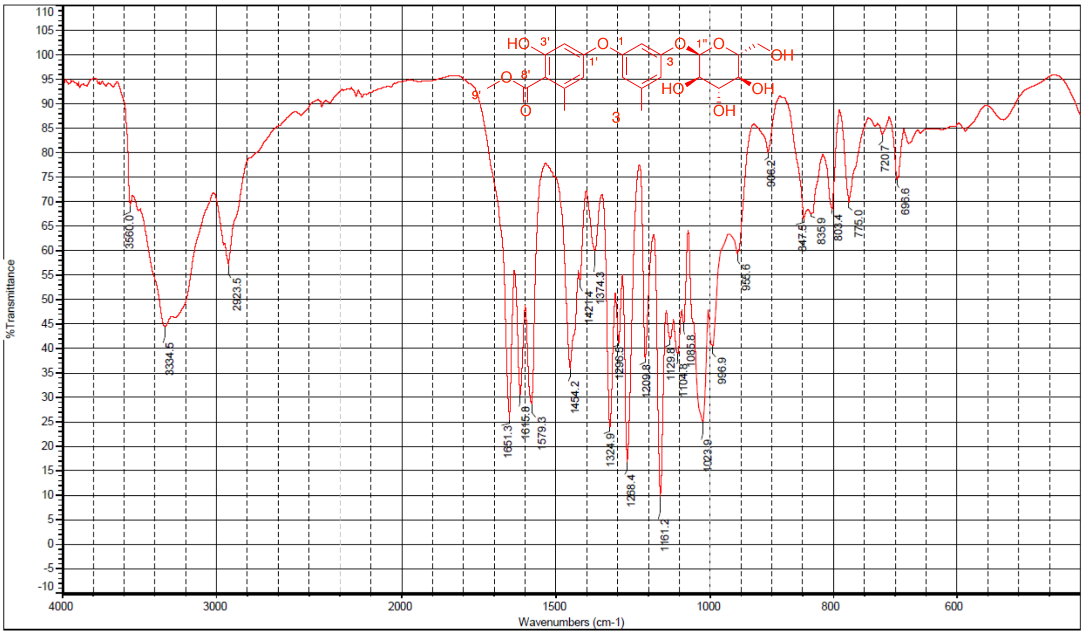

Figure S17. IR spectrum of compound **3**

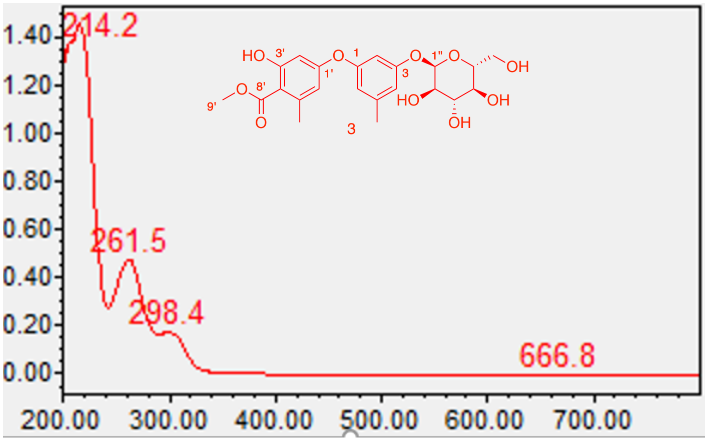

Figure S18. UV spectrum of compound **3**

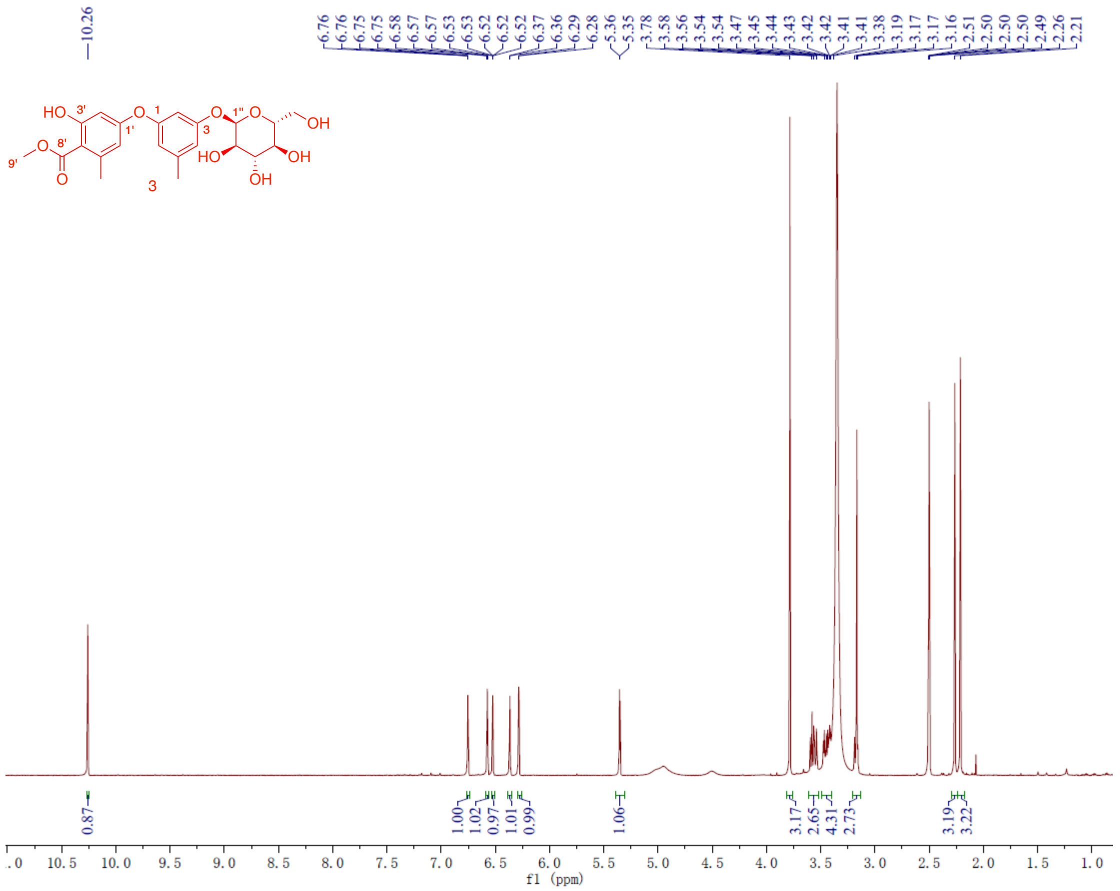

Figure S19. ^{1}H NMR spectrum of compound **3** in DMSO-d_6 (600 MHz)

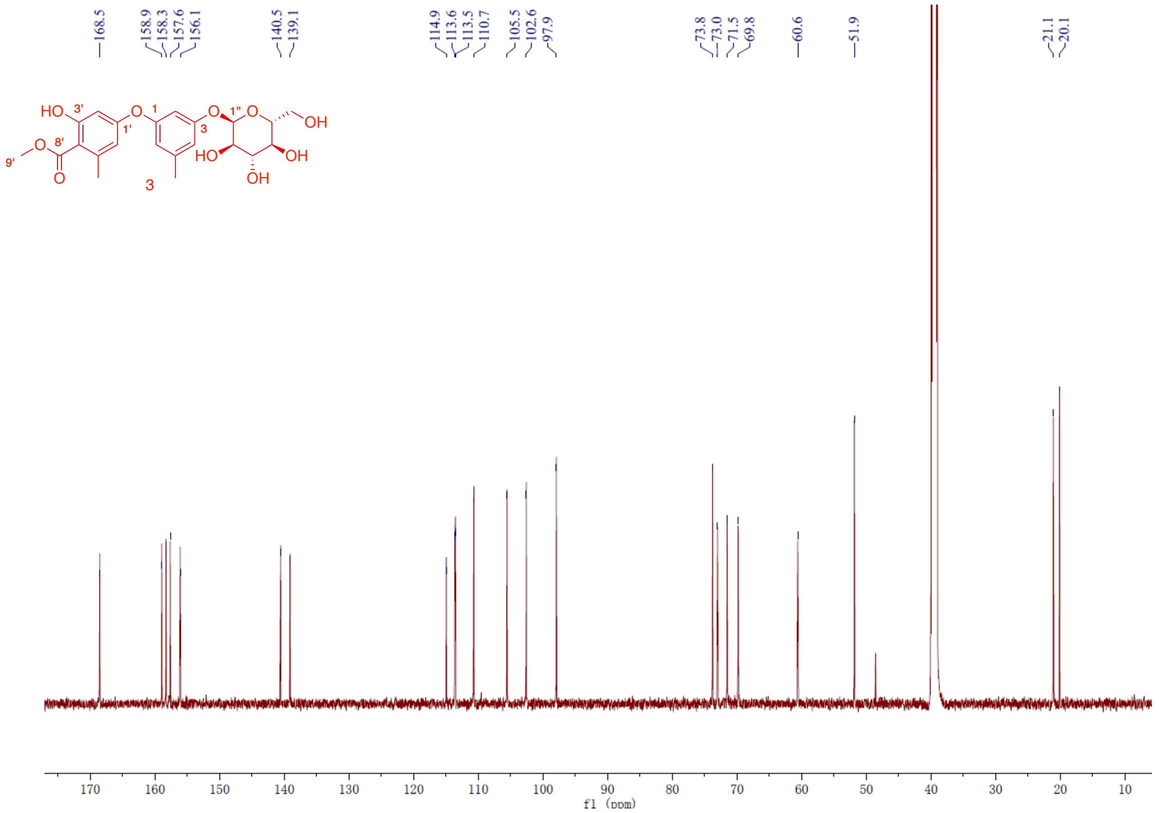

Figure S20. ^{13}C NMR spectrum of compound **3** in DMSO-d_6 (150 MHz)

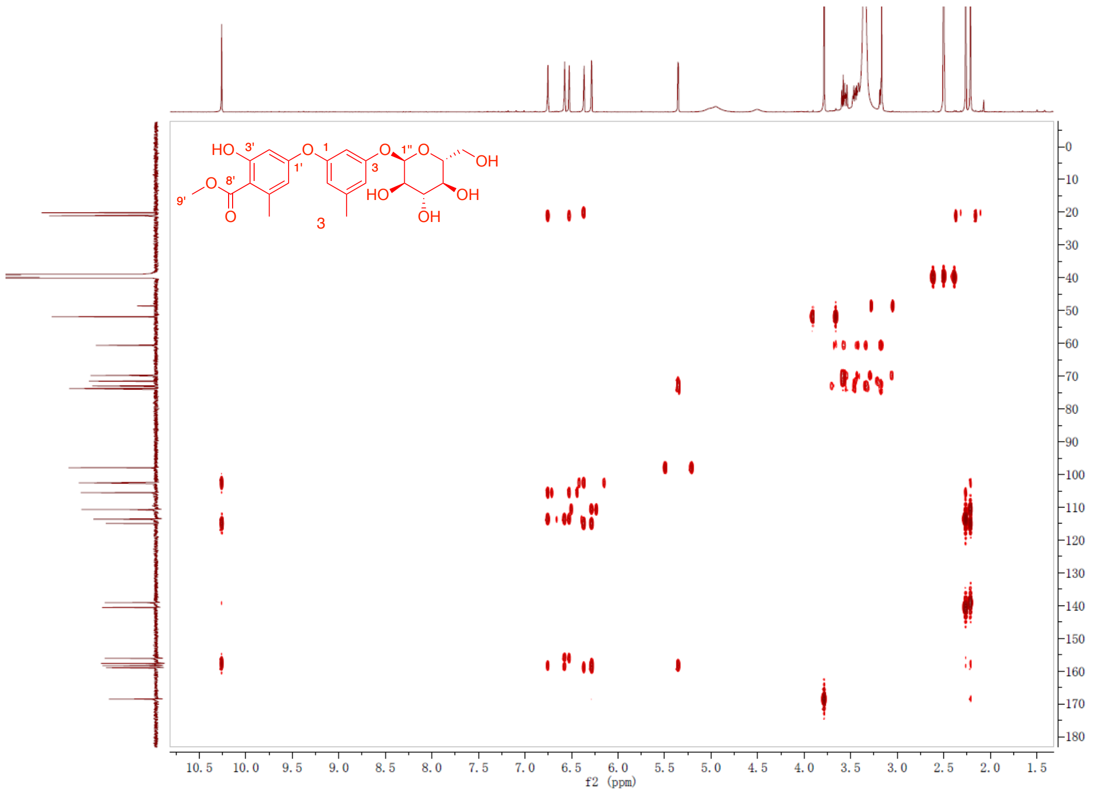

Figure S21. HMBC spectrum of compound **3** in DMSO-*d*6

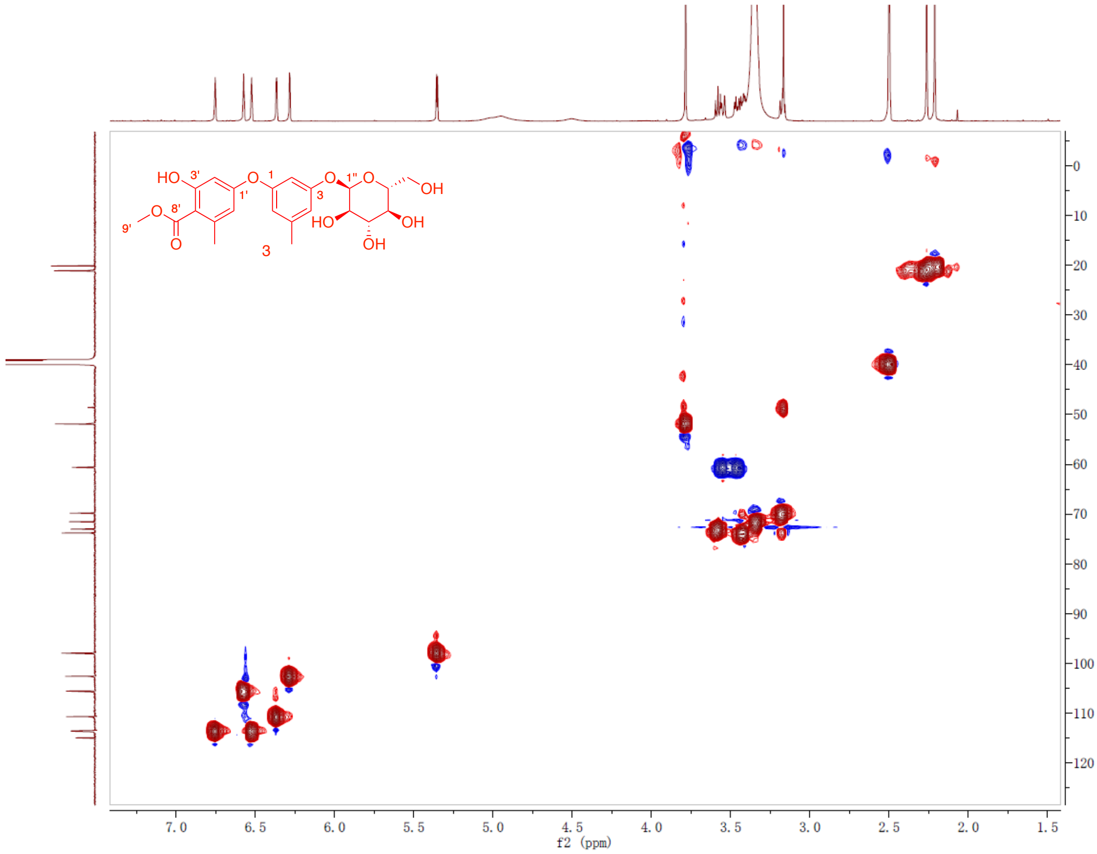

Figure S22. HSQC spectrum of compound **3** in DMSO-*d*6

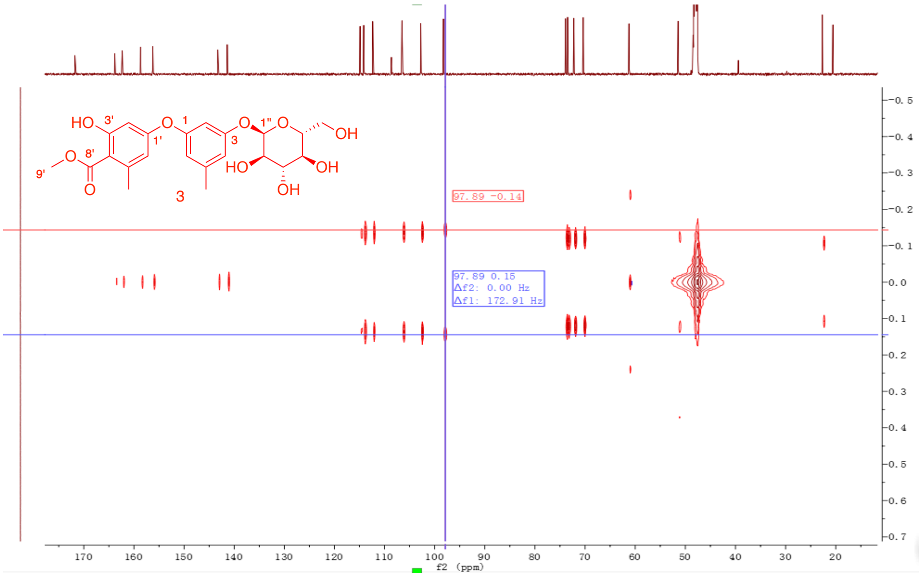

Figure S23. J Resolved HSQC spectrum of compound **3** in CD_3OD

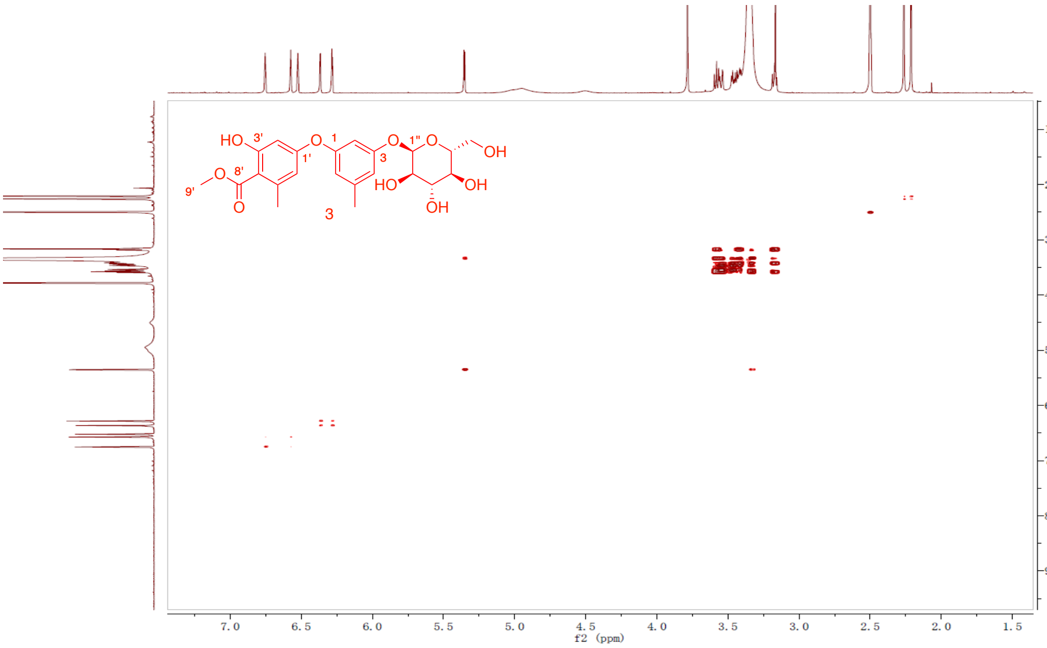

Figure S24. COSY spectrum of compound **3** in DMSO-d_6

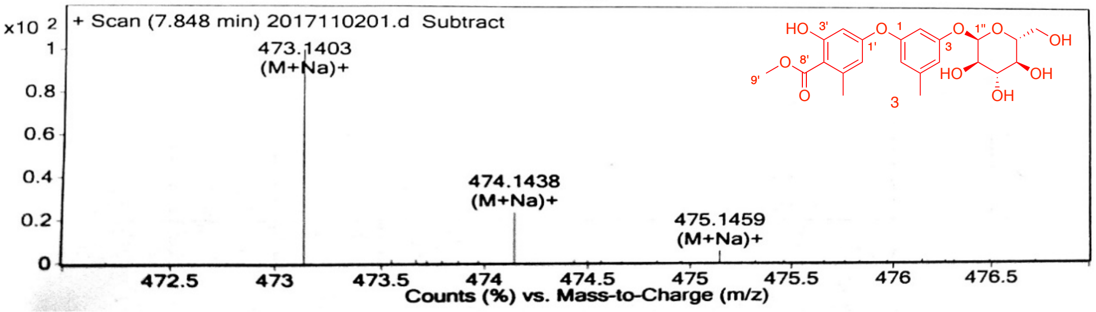

m/z	Ion	Formula	Abundance
473.1403	(M+Na)+	C22 H26 Na O10	207149.1

Best	Formula (M)	Ion Formula	Score	Cross Sco	Mass	Calc Mass	Calc m/z	Diff (ppm)	Abs Diff (ppm)	Mass Match	Abund Match	Spacing Match	DBE
☑	C22 H26 O10	C22 H26 Na O10	99.79		450.151	450.1526	473.1418	3.46	3.46	99.63	99.88	100	10

Figure S25. HRESIMS of compound **3**

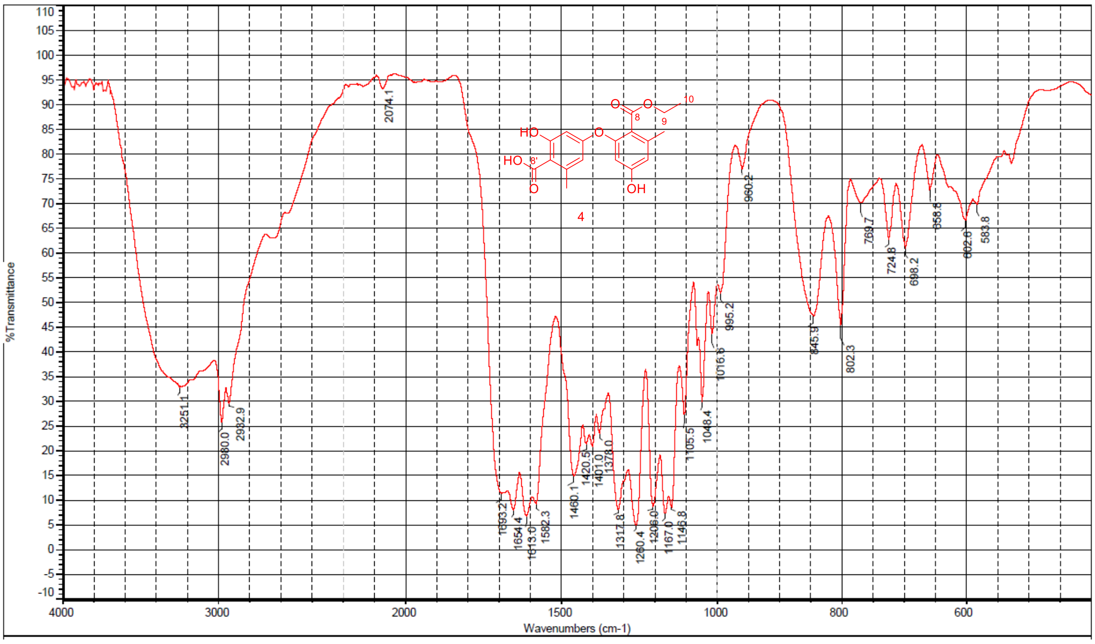

Figure S26. IR spectrum of compound **4**

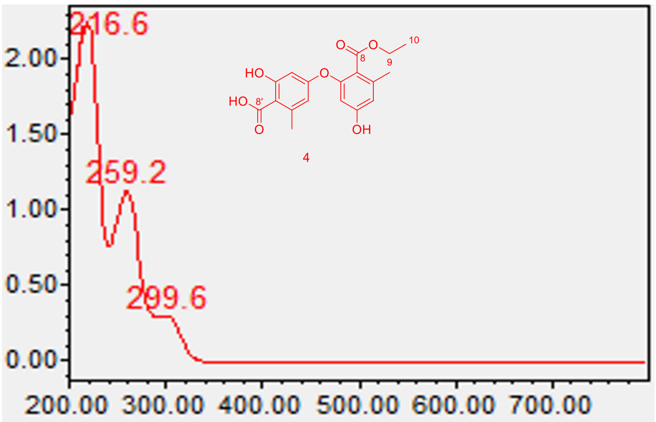

Figure S27. UV spectrum of compound **4**

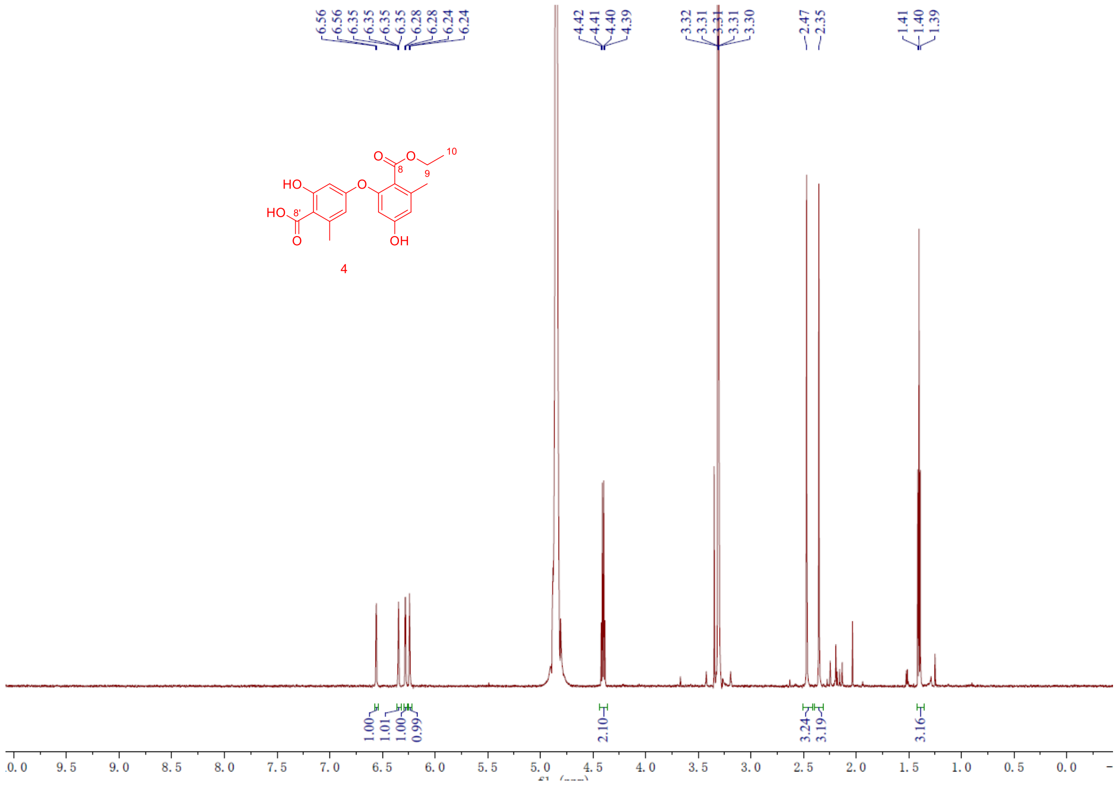

Figure S28. ^{1}H NMR spectrum of compound **4** in CD_3OD (600 MHz)

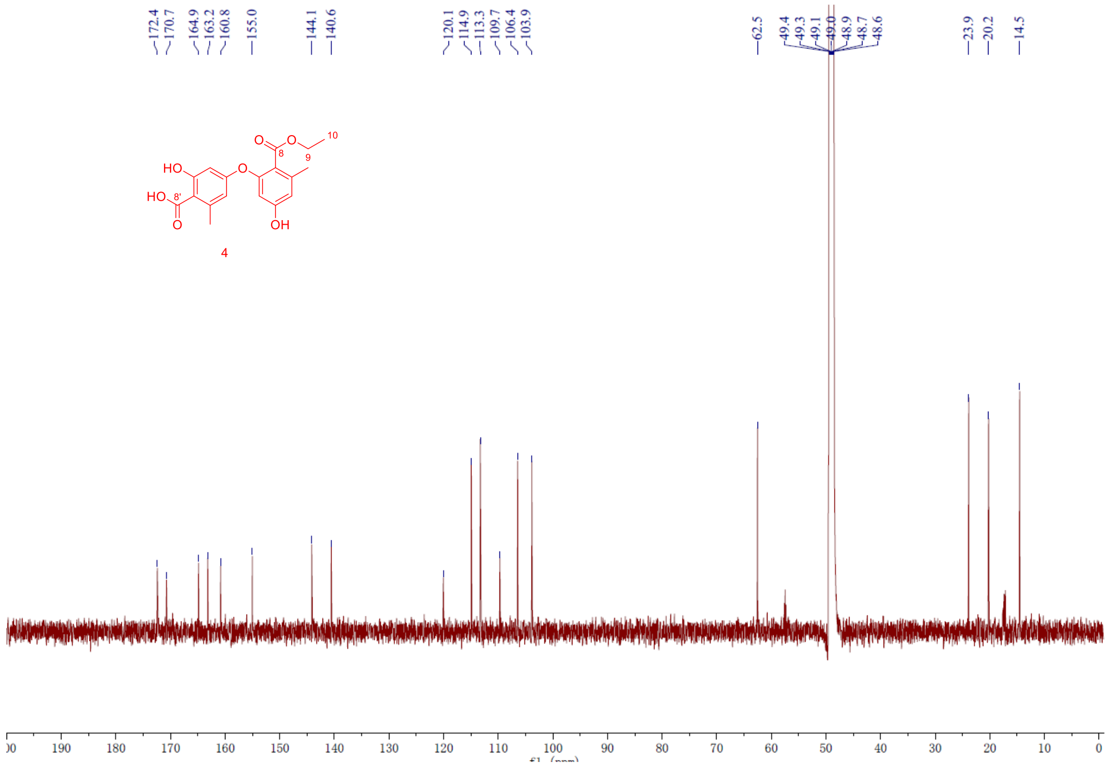

Figure S29. ^{13}C NMR spectrum of compound **4** in CD_3OD (150 MHz)

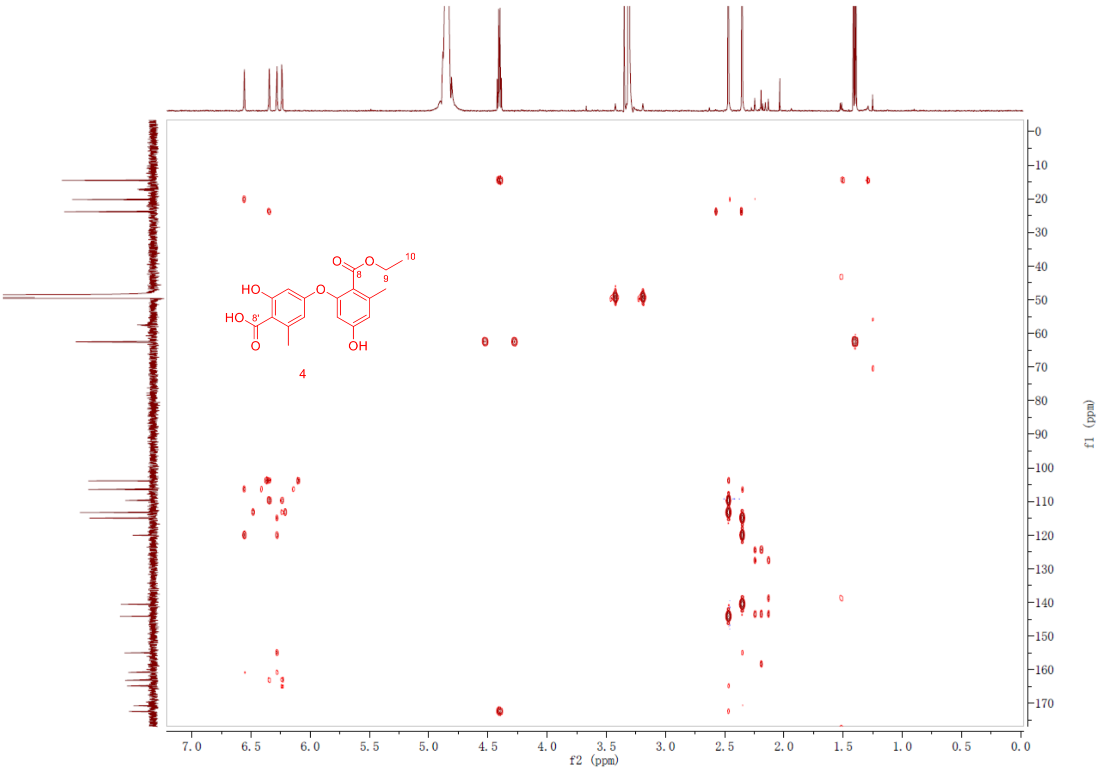

Figure S30. HMBC spectrum of compound **4** in CD3OD

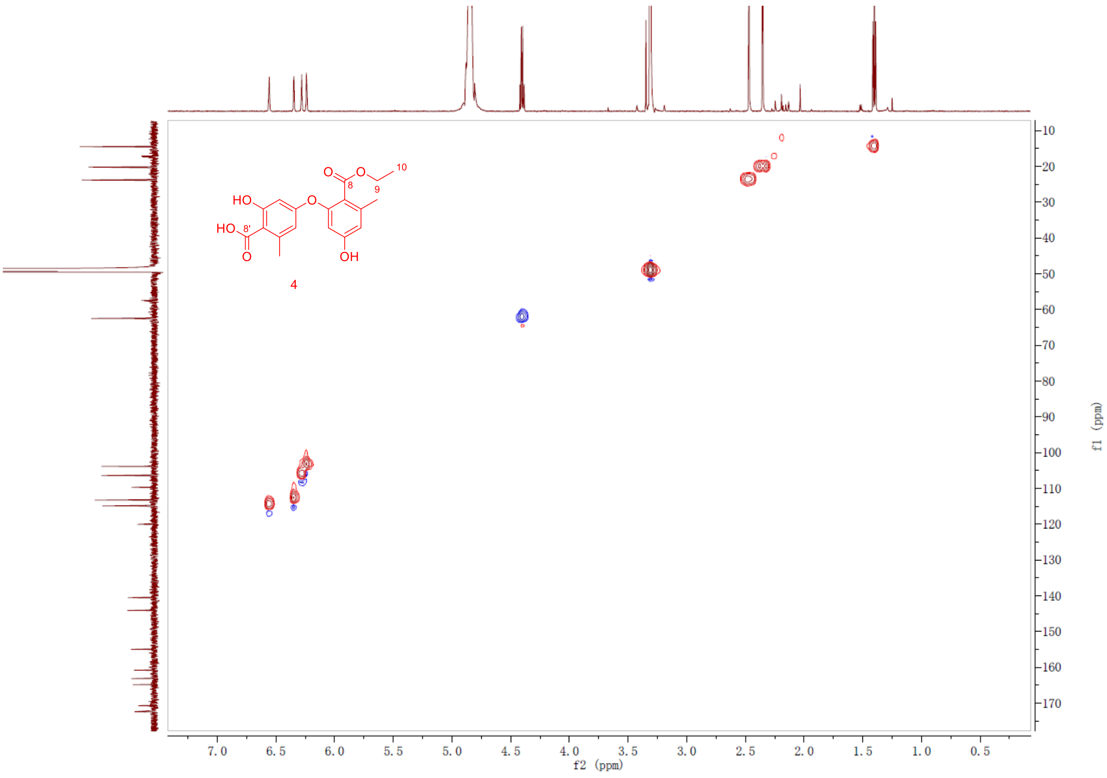

Figure S31. HSQC spectrum of compound **4** in CD3OD

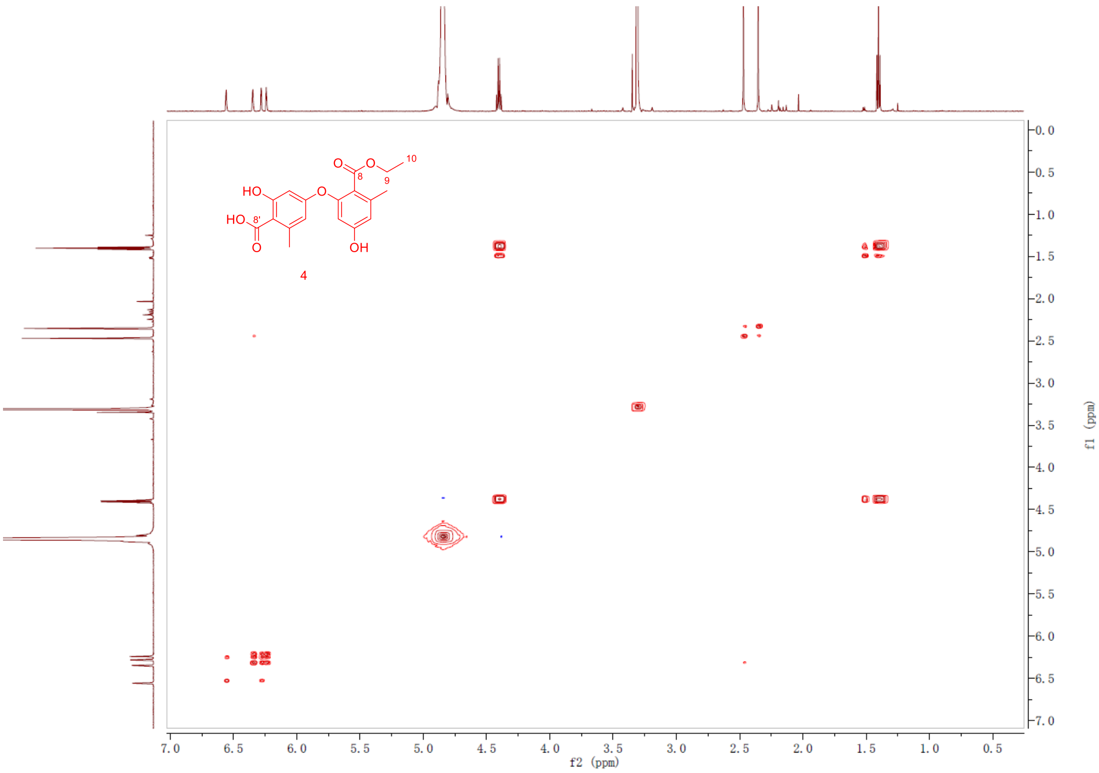

Figure S32. COSY spectrum of compound **4** in CD3OD

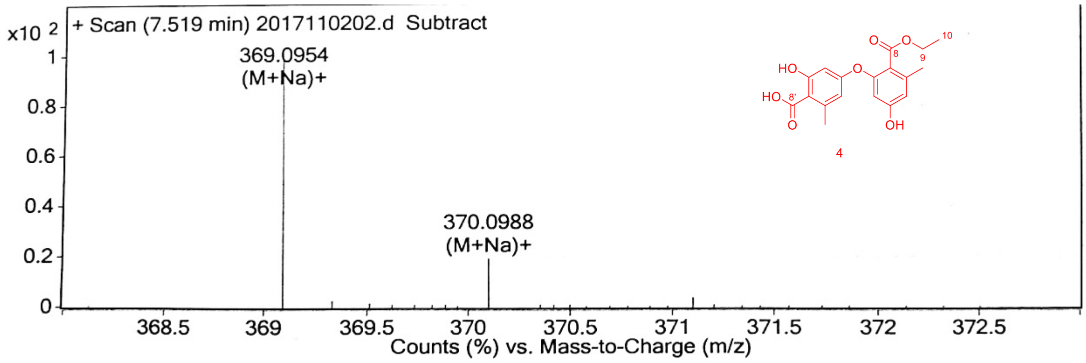

m/z	Ion	Formula	Abundance
369.0954	(M+Na)+	C18 H18 Na O7	85544.7

Best	Formula (M)	Ion Formula	Score	Cross Sco	Mass	Calc Mass	Calc m/z	Diff (ppm)	Abs Diff (ppm)	Mass Match	Abund Match	Spacing Match	DBE
☑	C18 H18 O7	C18 H18 Na O7	99.88		346.1062	346.1053	369.0945	-2.61	2.61	99.81	99.94	99.94	1

Figure S33. HRESIMS of compound **4**

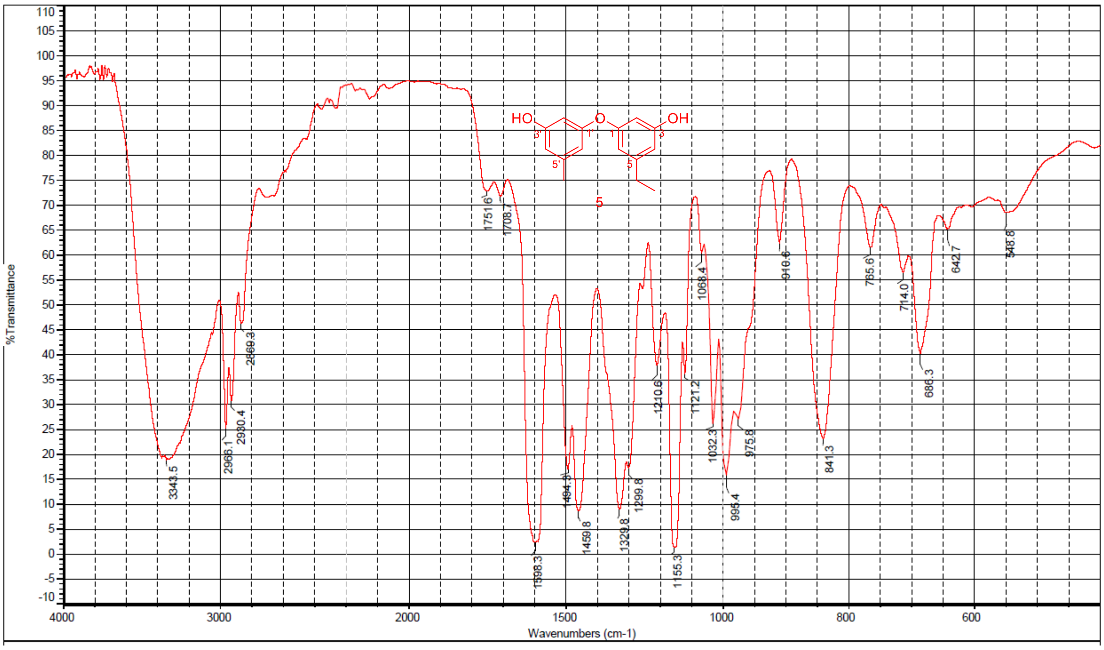

Figure S34. IR spectrum of compound **5**

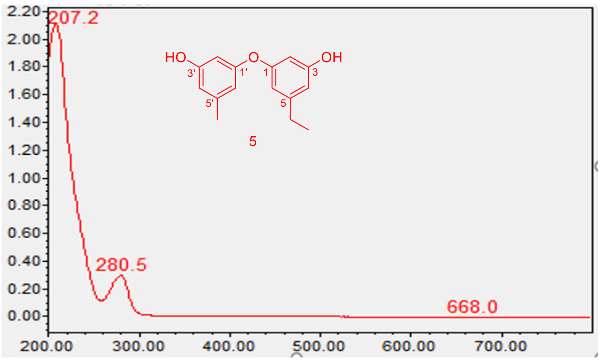

Figure S35. UV spectrum of compound **5**

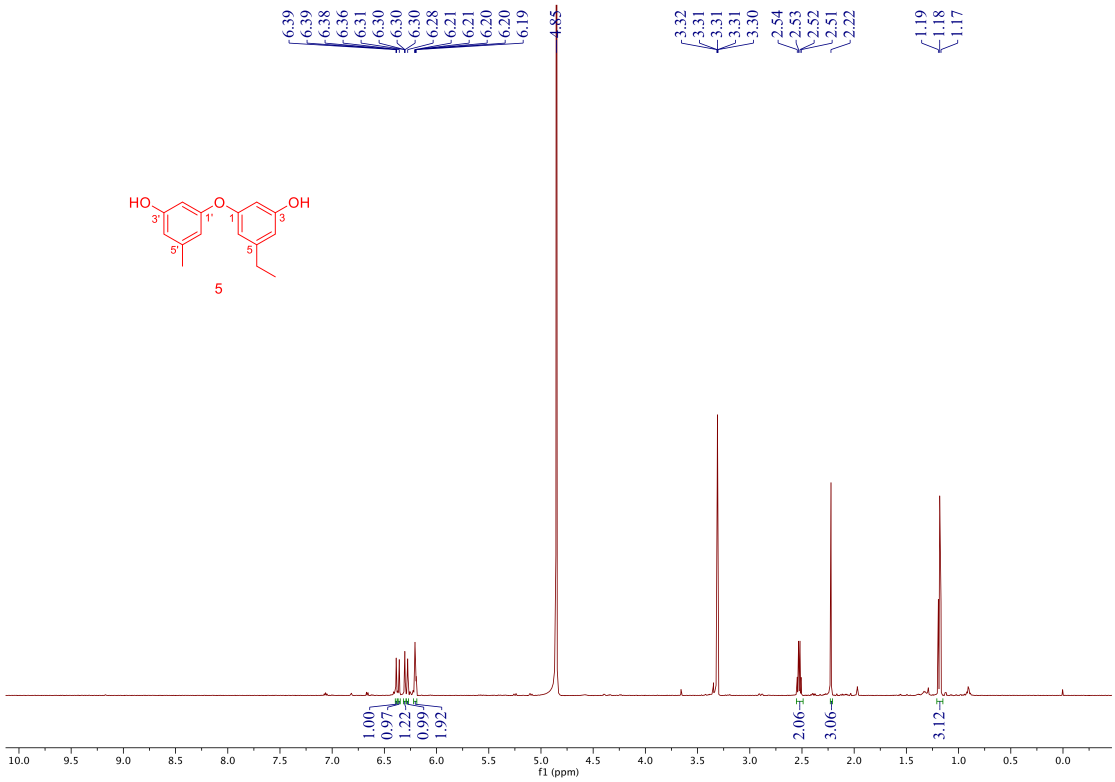

Figure S36. ^{1}H NMR spectrum of compound **5** in CD_3OD (600 MHz)

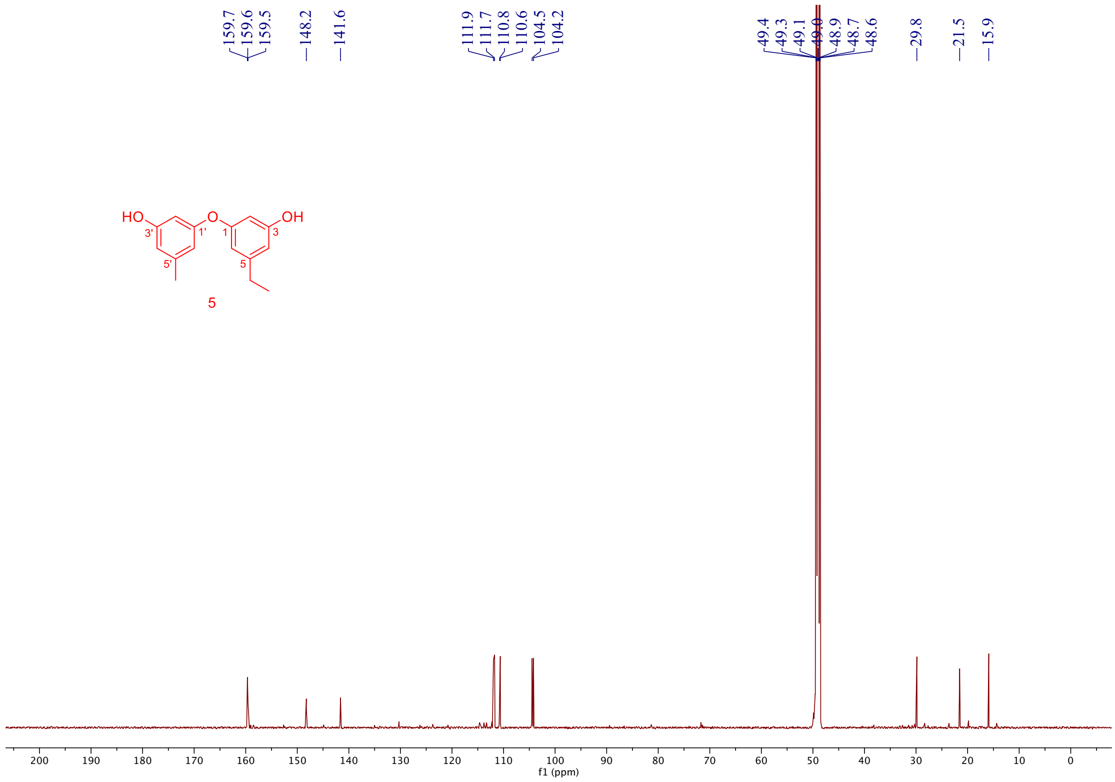

Figure S37. ^{13}C NMR spectrum of compound **5** in CD_3OD (150 MHz)

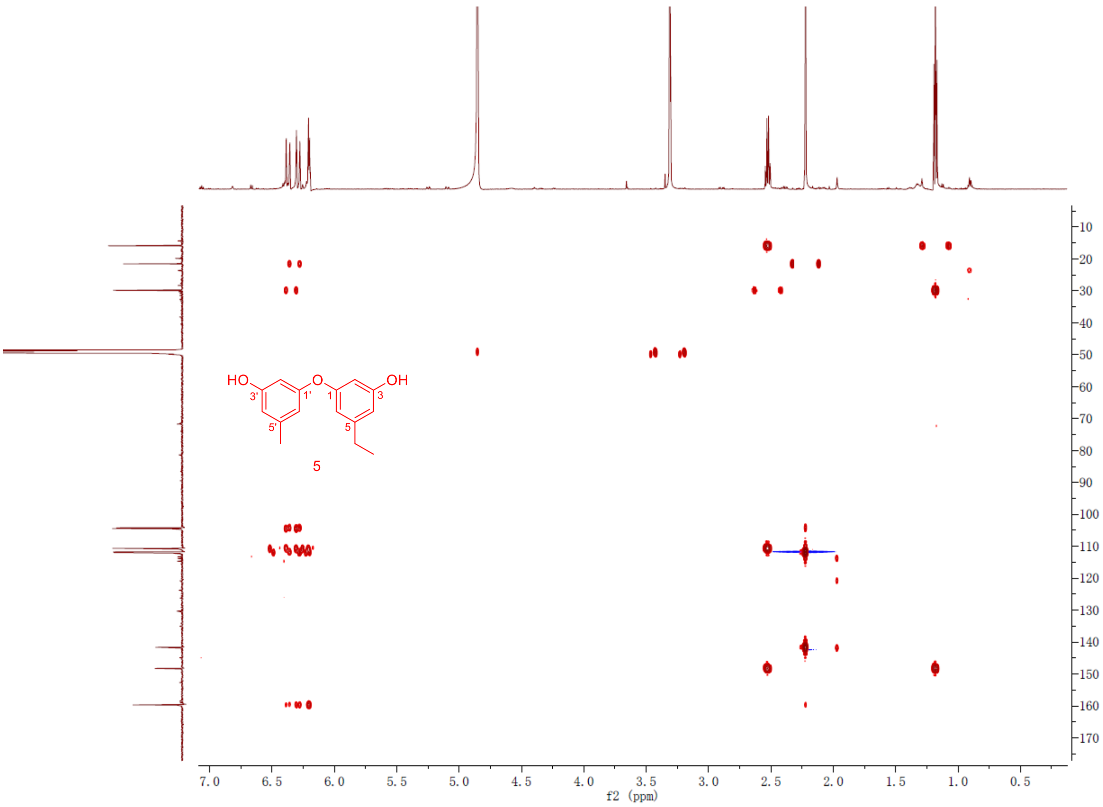

Figure S38. HMBC spectrum of compound **5** in CD3OD

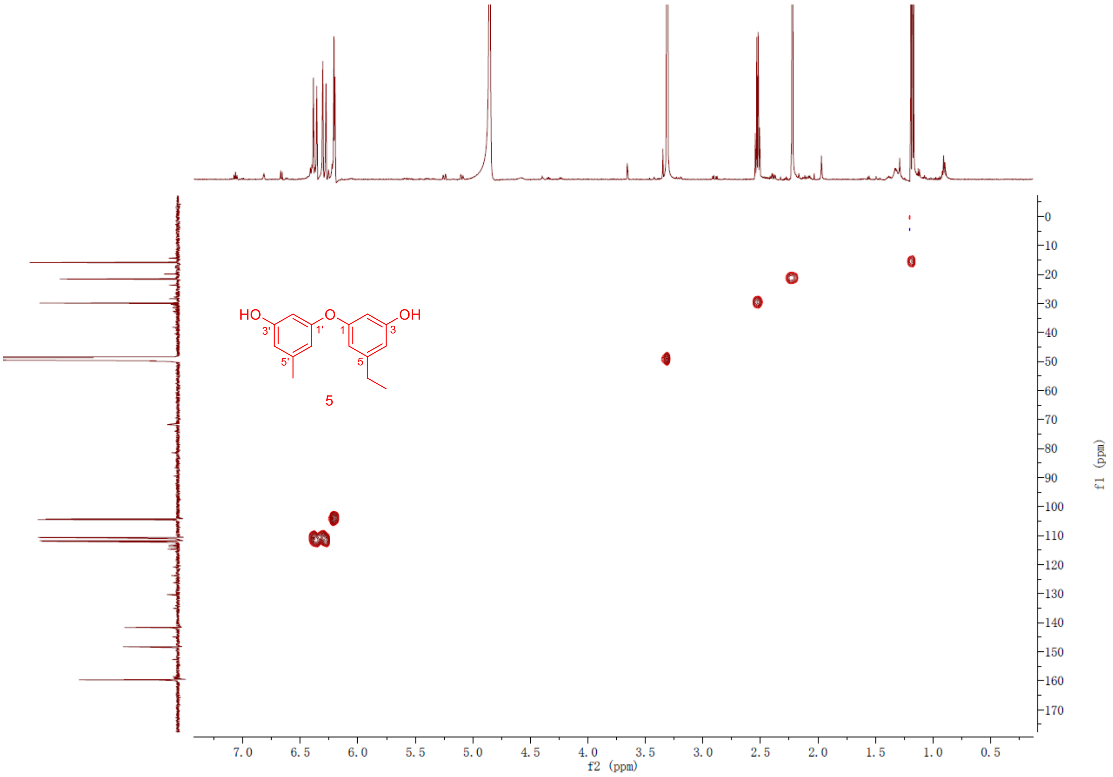

Figure S39. HSQC spectrum of compound **5** in CD3OD

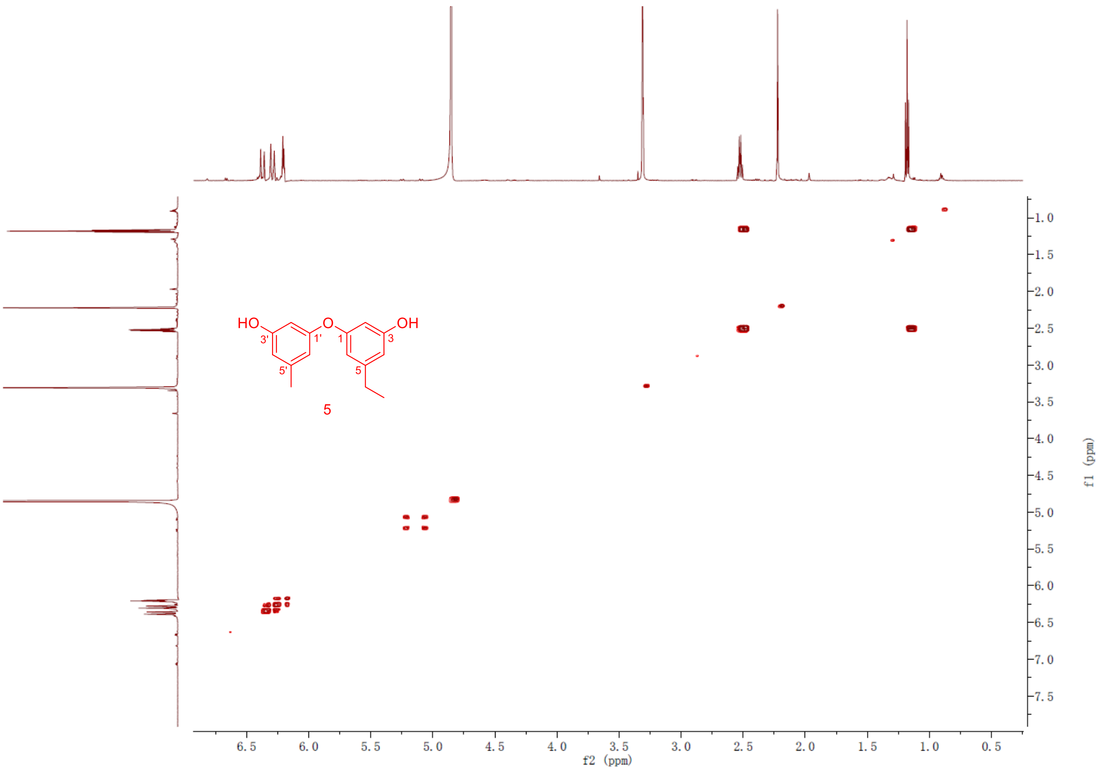

Figure S40. COSY spectrum of compound **5** in CD3OD

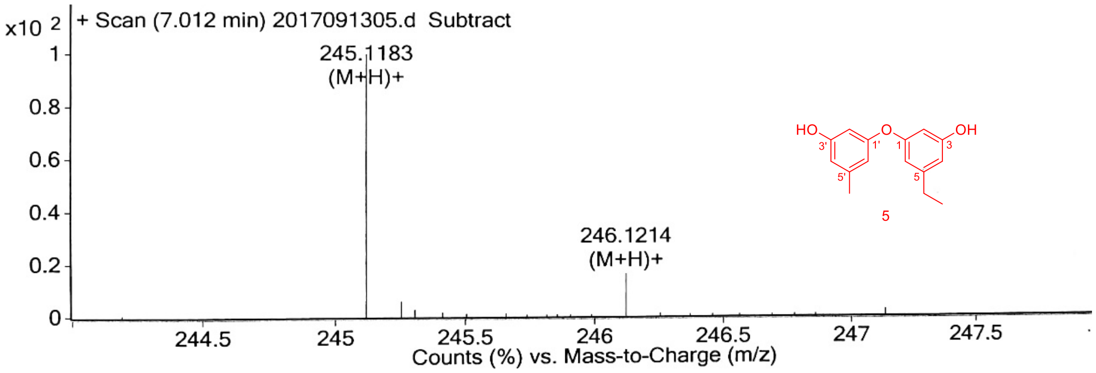

m/z	Ion	Formula	Abundance
245.1183	(M+H)+	C15 H17 O3	629669.1

Best	Formula (M)	Ion Formula	Score	Cross Sco	Mass	Calc Mass	Calc m/z	Diff (ppm)	Abs Diff (ppm)	Mass Match	Abund Match	Spacing Match	DBE
☑	C15 H16 O3	C15 H17 O3	99.49		244.111	244.1099	245.1172	-4.28	4.28	99.46	99.94	99.01	8

Figure S41. HRESIMS of compound **5**

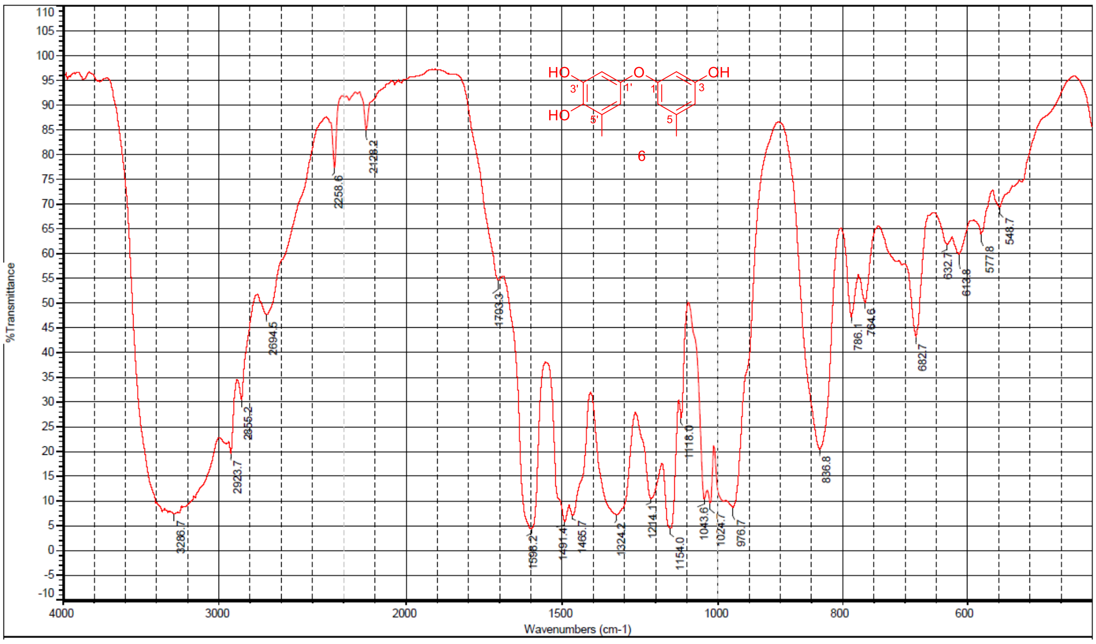

Figure S42. IR spectrum of compound **6**

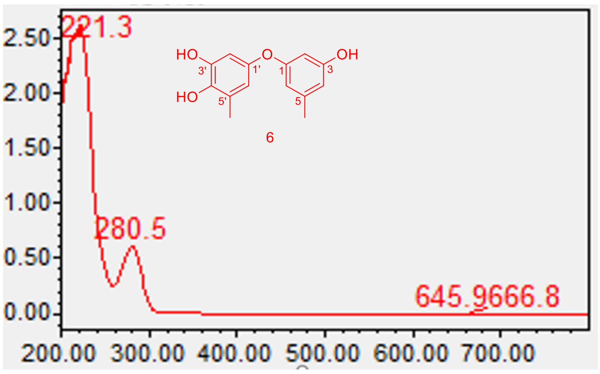

Figure S43. UV spectrum of compound **6**

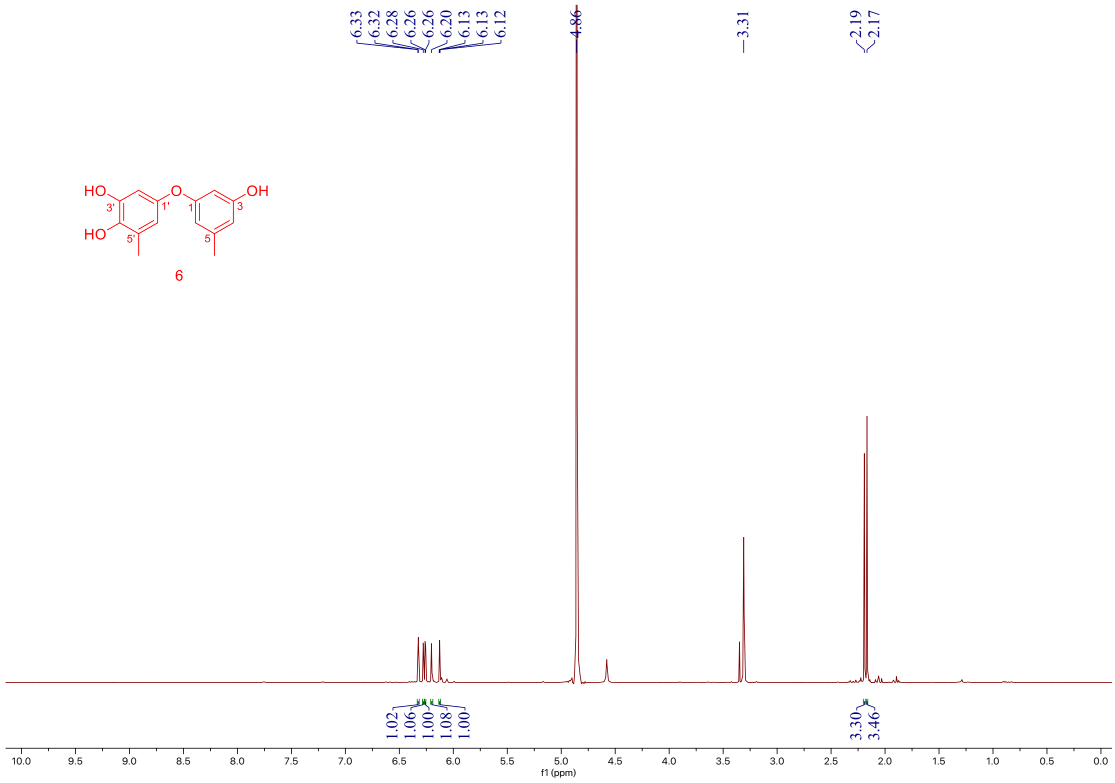

Figure S44. ^{1}H NMR spectrum of compound **6** in CD_3OD (600 MHz)

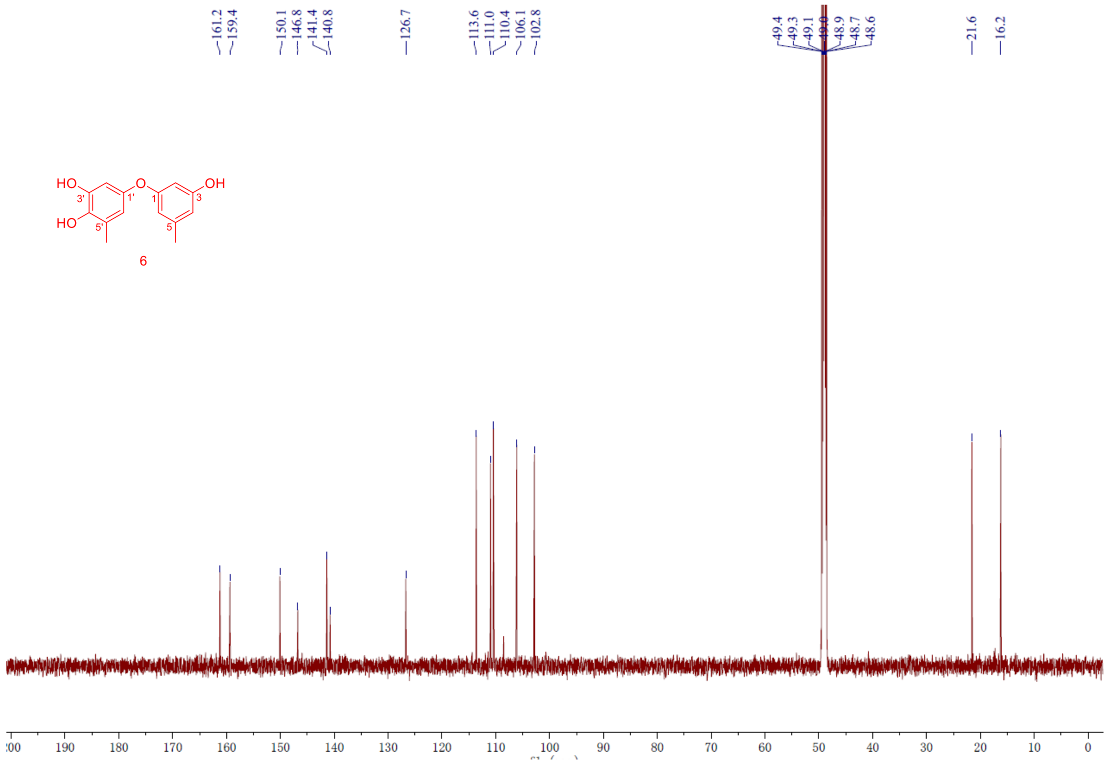

Figure S45. ^{13}C NMR spectrum of compound **6** in CD_3OD (150 MHz)

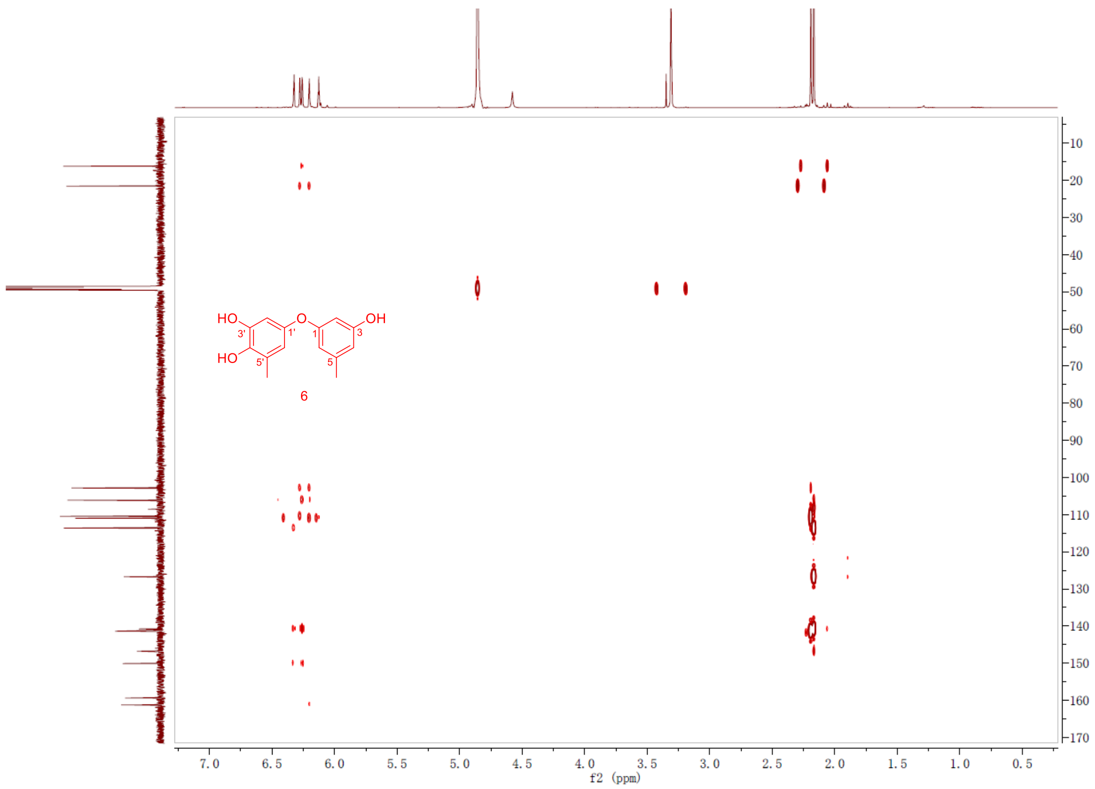

Figure S46. HMBC spectrum of compound **6** in CD3OD

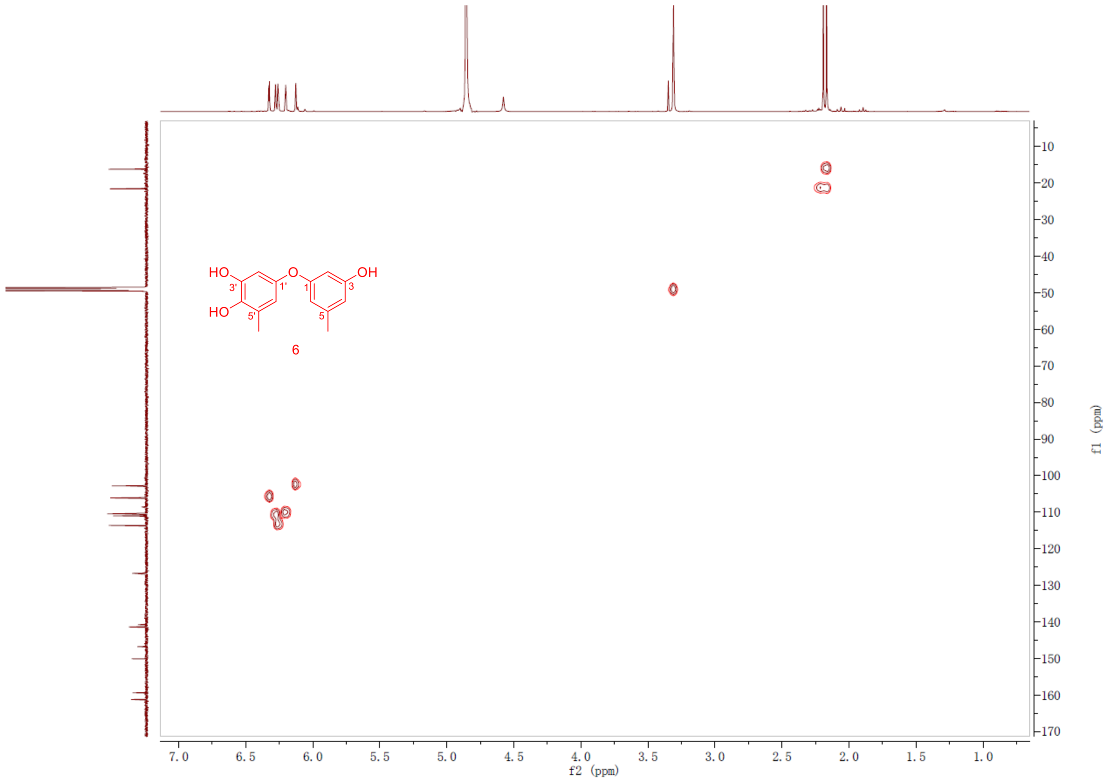

Figure S47. HSQC spectrum of compound **6** in CD3OD

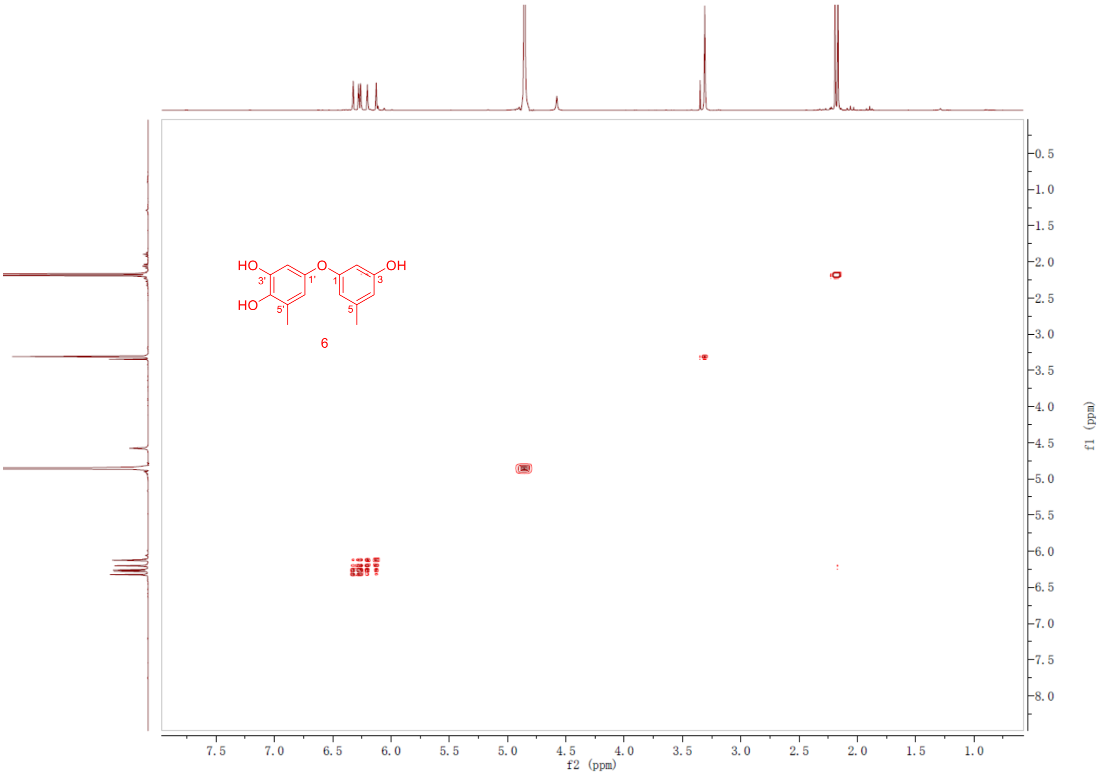

Figure S48. COSY spectrum of compound **6** in CD3OD

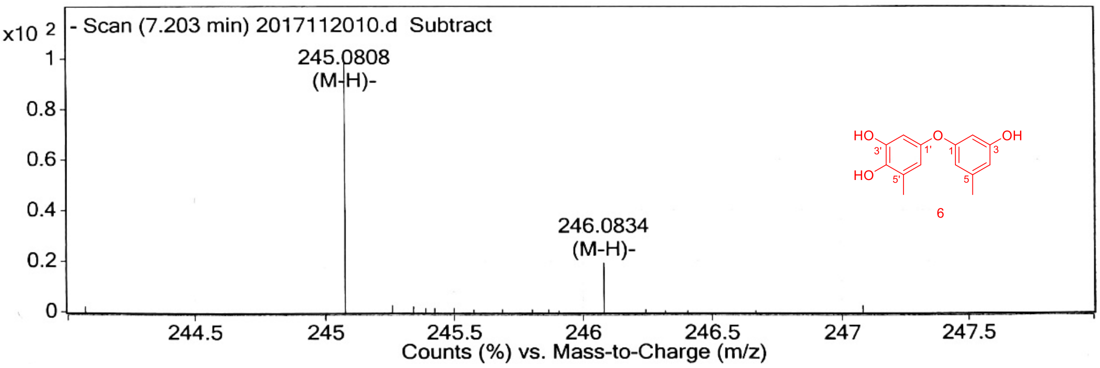

m/z	Ion	Formula	Abundance
245.0808	(M-H)-	C14 H13 O4	37664.6

Best	Formula (M)	Ion Formula	Score	Cross Sco	Mass	Calc Mass	Calc m/z	Diff (ppm)	Abs Diff (ppm)	Mass Match	Abund Match	Spacing Match	DBE
✓	C14 H14 O4	C14 H13 O4	99.06		246.088	246.0892	245.0819	4.73	4.73	99.34	98.04	99.73	8

Figure S49. HRESIMS of compound **6**

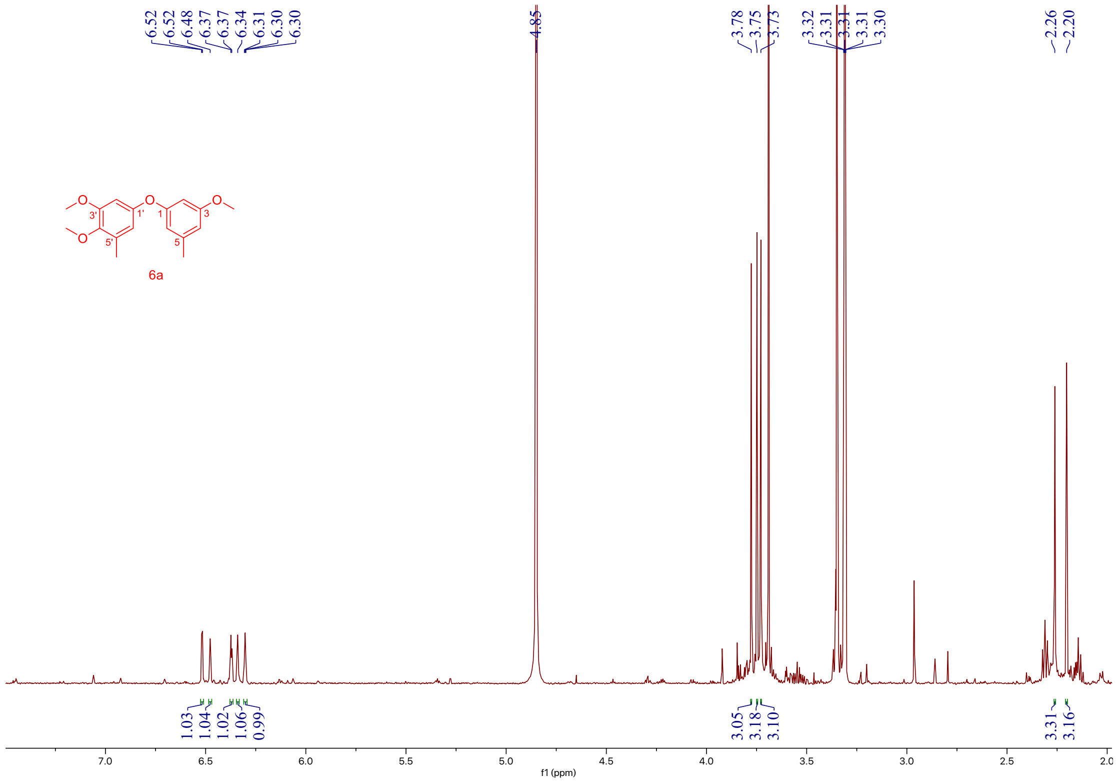

Figure S50. ^{1}H NMR spectrum of compound **6a** in CD_3OD (600 MHz)

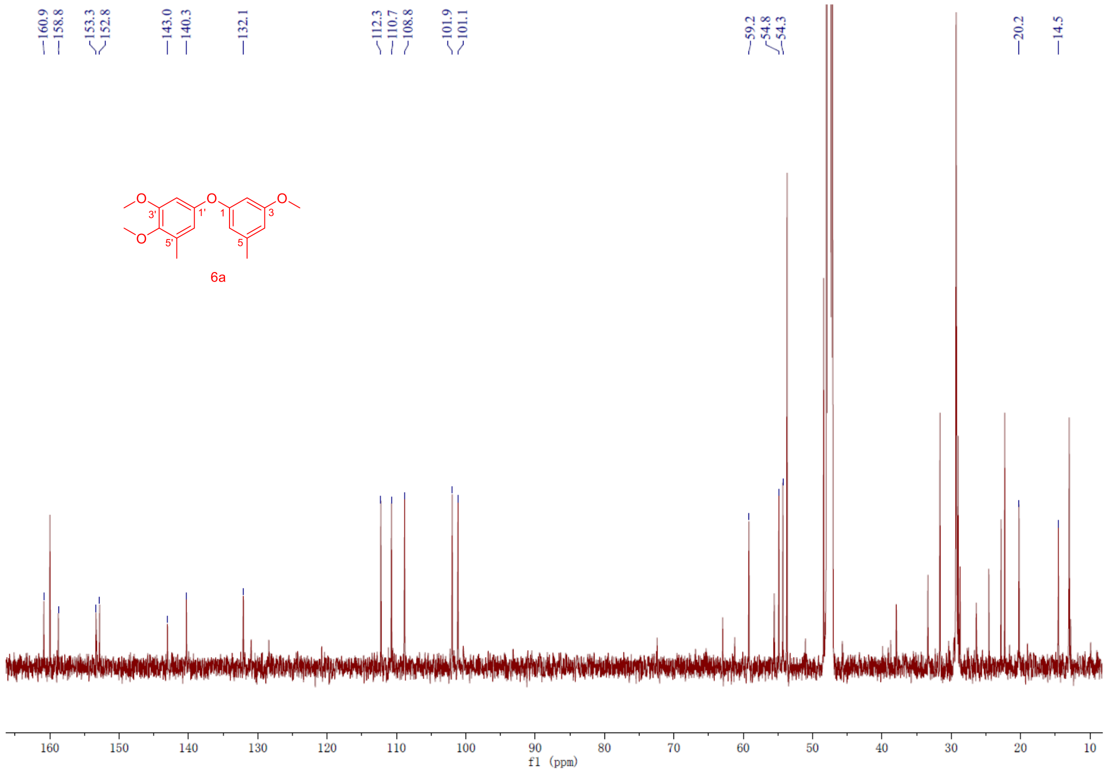

Figure S51. ^{13}C NMR spectrum of compound **6a** in CD_3OD (150 MHz)

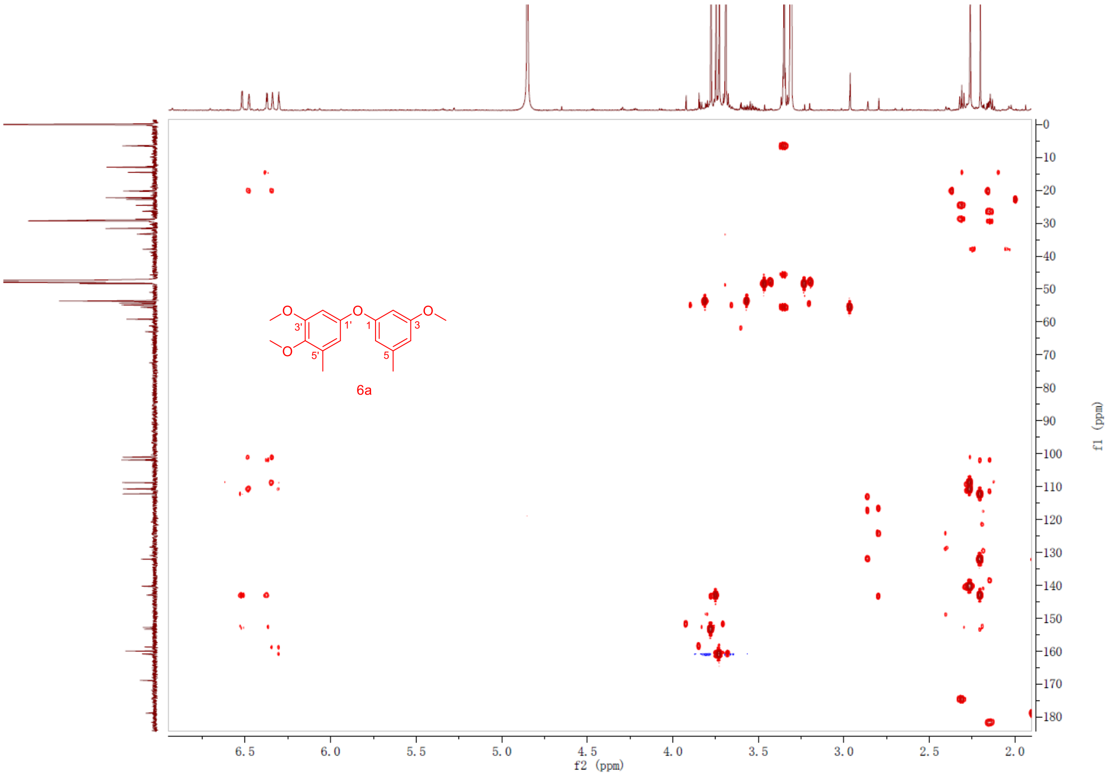

Figure S52. HMBC spectrum of compound **6a** in CD3OD

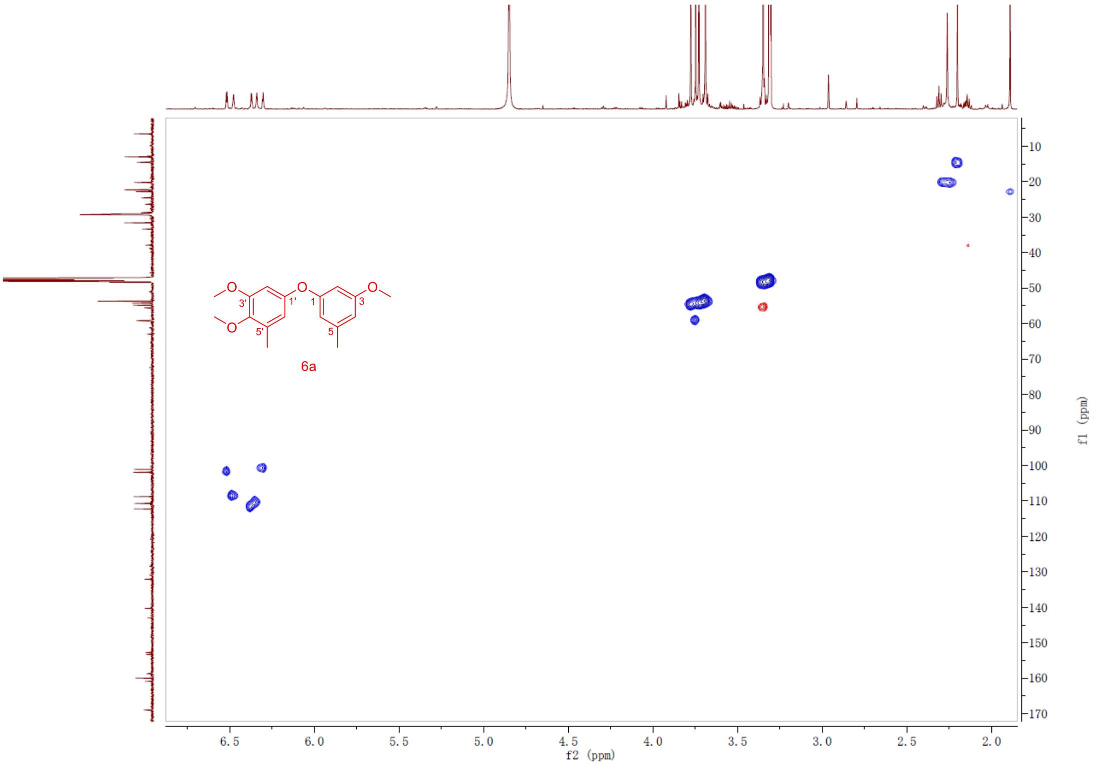

Figure S53. HSQC spectrum of compound **6a** in CD3OD

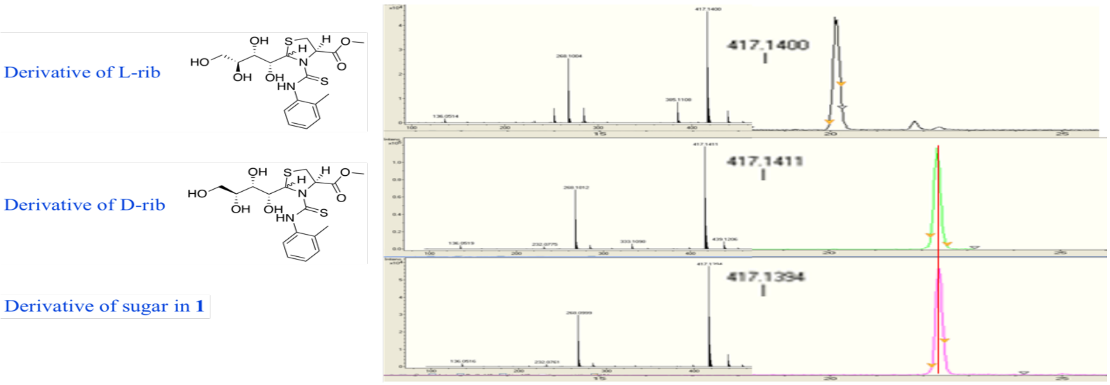

Figure S54. LC-ESI-MS analysis of derivative of sugar moiety in **1.**
The sugar moiety in **1** was determined as D-ribose by ESI mass spectrum (extraction ion chromatogram at *m*/*z* 417, t_R 20.1 for L-ribose, t_R 22.3 for D-ribose).

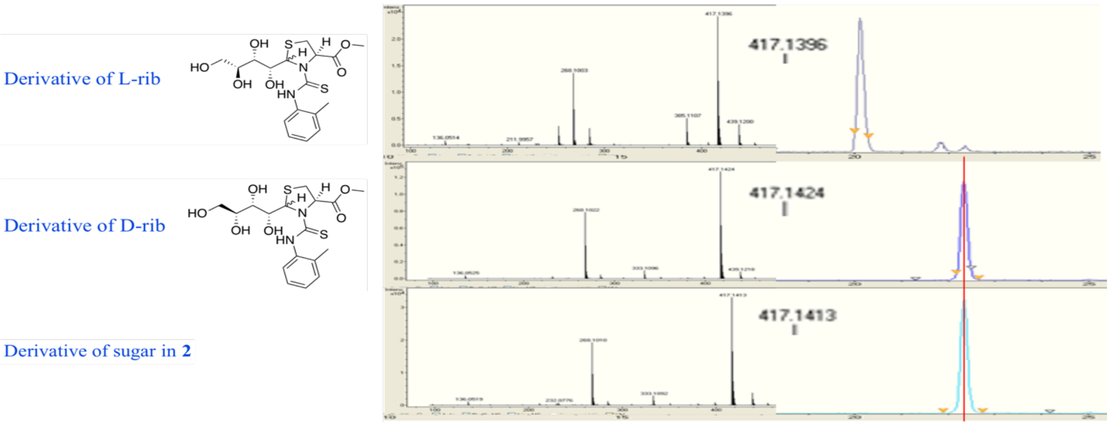

Figure S55. LC-ESI-MS analysis of derivatives of sugar moiety in **2.** The sugar moiety in **2** was determined as D-ribose by ESI mass spectrum (extraction ion chromatogram at *m*/*z* 417, t_R 20.1 for L-ribose, t_R 22.3 for D-ribose).

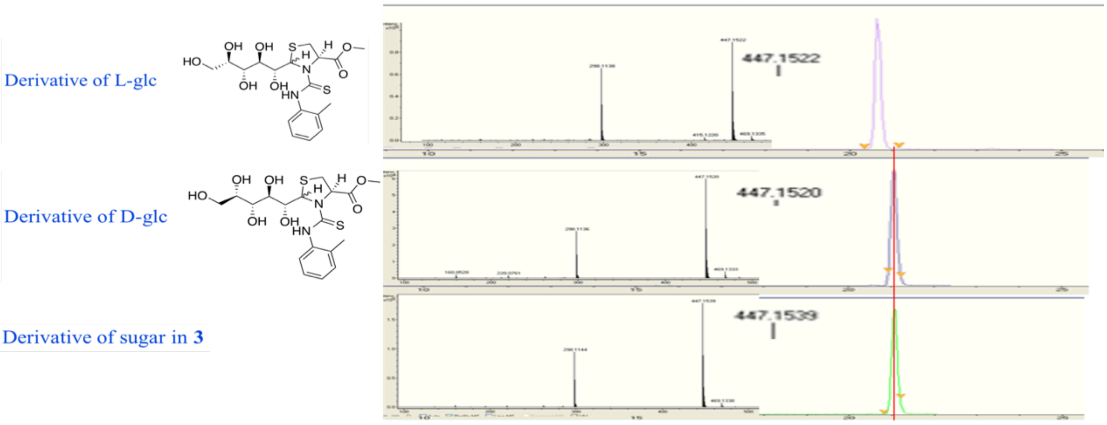

Figure S56. LC-ESI-MS analysis of derivatives of sugar moiety in **3.** The sugar moiety in **3** was determined as D-glucose by ESI mass spectrum (extraction ion chromatogram at *m*/*z* 447, t_R 20.6 for L-glucose, t_R 21.0 for D-glucose).

>TGCTCGTAGTGACCTGCGGAAGGATCATTACTGAGTGCGGGCTGCCTCCGGGCGCCCAACCTCC

CACCCGTGAATACCTAACACTGTTGCTTCGGCGGGGAGCTCCCTCGGGGGCGAGCCGCCGGGG

ACTACTGAACTTCATGCCTGAGAGTGATGCAGTCTGAGTCTGAATATAAAATCAGTCAAAACTTT

CAACAATGGATCTCTTGGTTCCGGCATCGATGAAGAACGCAGCGAACTGCGATAAGTAATGTGA

ATTGCAGAATTCAGTGAATCATCGAGTCTTTGAACGCACATTGCGCCCCCTGGCATTCCGGGGG

CATGCCTGTCCGAGCGTCATTGCTGCCCATCAAGCCCGGCTTGTGTGTTGGGTCGTCGTCCCCCC

CGGGGGACGGGCCCGAAAGGCAGCGGCGGCACCGTGTCCGGTCCTCGAGCGTATGGGGCTTTA

TCACCCGCTCGACTAGGGCCGGCCGGGCGCCAGCCGACGTCTCCAACCATTTTTCTTCAGGTTG

ACCTCGGATCAGGTAGGGATACCCGCTGAACTTAAGCATATCAATAAGCGGAGGAA//

Figure S57. The internal transcribed spacers (ITS) sequence of strain FNA026
